# Phylodynamics of H1N1/2009 influenza reveals the transition from host adaptation to immune-driven selection

**DOI:** 10.1038/ncomms8952

**Published:** 2015-08-06

**Authors:** Yvonne C. F. Su, Justin Bahl, Udayan Joseph, Ka Man Butt, Heidi A. Peck, Evelyn S. C. Koay, Lynette L. E. Oon, Ian G. Barr, Dhanasekaran Vijaykrishna, Gavin J. D. Smith

**Affiliations:** 1 Program in Emerging Infectious Diseases, Duke-NUS Graduate Medical School, 8 College Road, Singapore 169857, Singapore; 2 Division of Epidemiology, Human Genetics and Environmental Sciences, School of Public Health, The University of Texas, Houston, Texas 77030, USA; 3 World Health Organisation Collaborating Centre for Reference and Research on Influenza, Melbourne, Victoria 3000, Australia; 4 Molecular Diagnosis Centre, Department of Laboratory Medicine, National University Hospital, Singapore 119074, Singapore; 5 Department of Pathology, Singapore General Hospital, Singapore 169608, Singapore; 6 Department of Microbiology, Yong Loo Lin School of Medicine, National University of Singapore, Singapore 117597, Singapore; 7 Duke Global Health Institute, Duke University, Durham, North Carolina 27708, USA

## Abstract

Influenza A H1N1/2009 virus that emerged from swine rapidly replaced the previous seasonal H1N1 virus. Although the early emergence and diversification of H1N1/2009 is well characterized, the ongoing evolutionary and global transmission dynamics of the virus remain poorly investigated. To address this we analyse >3,000 H1N1/2009 genomes, including 214 full genomes generated from our surveillance in Singapore, in conjunction with antigenic data. Here we show that natural selection acting on H1N1/2009 directly after introduction into humans was driven by adaptation to the new host. Since then, selection has been driven by immunological escape, with these changes corresponding to restricted antigenic diversity in the virus population. We also show that H1N1/2009 viruses have been subject to regular seasonal bottlenecks and a global reduction in antigenic and genetic diversity in 2014.

## Abstract (web summary)

Influenza A H1N1/2009 virus emerged from swine and rapidly replaced the seasonal H1N1 virus. Here, the authors show that natural selection acting on H1N1/2009 after introduction into humans was driven by adaptation to the new host but later selection has been driven by immunological escape.

## Graphical abstract

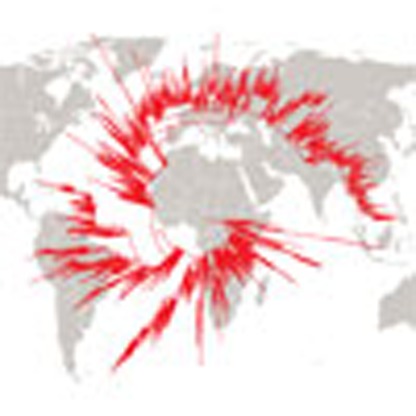

The influenza A(H1N1)pdm2009 (H1N1/2009) virus that emerged in humans during March and early April 2009 in the Americas spread rapidly among humans to develop into the first human influenza pandemic in over 40 years^1^,^2^,^3^. The initial transmission and spread of the H1N1/2009 virus was rapid with 168 countries reporting infections by July 2009, with some 162,300 laboratory-confirmed cases and over 1,100 human deaths^4^,^5^,^6^. Subsequently, it was estimated that >123,000 global deaths from March to December 2009 were potentially associated with H1N1/2009 infection, with a greater mortality impact observed in parts of the Americas compared with Europe and Australia^7^. Following this period, the H1N1/2009 virus has subsequently caused seasonal epidemics and has co-circulated with influenza A H3N2 and influenza B viruses in most countries^8^,^9^,^10^,^11^,^12^.

The emergence and circulation of H1N1/2009 virus contradicted prevailing theories at the time of pandemic emergence, wherein it was thought that a heterologous haemagglutinin (HA) subtype was required to initiate a new pandemic^13^. In contrast, the progenitors of the *HA* gene of the emergent H1N1/2009 virus that had been circulating in swine herds for at least 80 years before its emergence in humans^14^,^15^,^16^ had sufficient genetic and antigenic differentiation between the emergent strains and circulating human seasonal H1N1 viruses to cause a pandemic. Furthermore, the remaining gene segments of the H1N1/2009 virus, which were ultimately derived from avian, human and swine viruses, had been circulating in pigs for more than 20 years such that the human population manifested little or no pre-existing antigenic protection against these gene products^3^.

The emergence of the H1N1/2009 virus resulted in the rapid replacement of seasonal H1N1 virus that had circulated in humans for over 70 years (from 1918 to 1957 and from 1977 to 2009) (ref. 13); however, the mechanisms behind such influenza strain replacement in humans are still not well understood. Pre-adaptation of the H1N1/2009 *HA* gene to mammalian hosts through circulation in swine for several decades may have contributed to their emergence in humans^17^; however, differences in host biology between humans and swine likely affected the early adaptive evolution of H1N1/2009 virus in humans, which remains to be characterized. Interestingly, the A/California/7/2009-like H1N1/2009 virus has been the recommended World Health Organization (WHO) vaccine strain for inclusion into the seasonal influenza vaccine for both the Southern and Northern Hemisphere recommendations from 2010 to 2016, indicating that this lineage has not undergone significant antigenic change despite causing several seasonal influenza epidemics^18^.

During the initial weeks of the pandemic, estimates of the basic reproductive ratio (*R*_0_)—the average number of secondary infections produced by an infection and is an indicator of the pathogen's transmission potential—was 1.12 (95% Bayesian confidence interval: 1.07–1.16) in North America^19^. In contrast, country-specific estimates of the median *R*_0_ from the Southern Hemisphere ranged from 1.2 to 1.8 (ref. 20). A more recent review^21^ summarized the reported *R*_0_ estimates for H1N1/2009 viruses from 57 published regional articles (for example, Australia^22^, Canada^23^, Chile^24^, Hong Kong^25^, Italy^12^, Netherlands^26^ and South Africa^27^), indicating that the median *R*_0_ ranged from 1.3 to 1.7 in the first year. Notably, these *R*_0_ estimates showed a significant association with the percentage of children in different populations, wherein a population with more children had a higher *R*_0_ estimate^20^. This is reflected in epidemiological findings indicating that children and adolescents (<20 years old) were more susceptible to H1N1/2009 infections than adults (≥20 years old), highlighting that adults may have had a greater pre-existing immunity against the virus^1^,^20^. The *R*_0_ estimates of the H1N1/2009 pandemic were, however, lower than the previous three pandemics which ranged from 1.4–5.4 for 1918 Spanish H1N1 (refs 28, 29, 30, 31), to 1.4–2.6 for 1957 H2N2 (refs 30, 32, 33) and 1.06–3.58 for 1968 H3N2 (refs 30, 34); but similar to typical human seasonal influenza estimates of 1.2–1.4 (ref. 35).

The 2009 influenza pandemic was the first to occur in the genomic era, with over 2,000 H1N1/2009 genomes made publically available from infections during 2009–2010 alone. This provided an unprecedented opportunity to understand the emergence and establishment of a novel pathogen in humans across various spatial and temporal scales. Although the early emergence and diversification of H1N1/2009 virus has been well described^4^,^5^,^8^, its ongoing evolution, including genetic and antigenic drift, reassortment and migration patterns have been poorly investigated. To investigate the global evolution of the H1N1/2009 virus, we analysed all publicly available H1N1/2009 sequence data along with an additional 214 full genomes generated from virological surveillance in Singapore from 2009 to 2014. Here we provide insight into the spatially and temporally heterogeneous evolutionary dynamics and migration of H1N1/2009 viruses as it has become entrenched as a human seasonal influenza virus. We detail changes in the natural selection acting on H1N1/2009 directly after introduction into humans, largely driven by adaptation to the new host, and later in the post-pandemic period where immune-driven selection was observed.

## Results

### Population genetics of H1N1/2009 viruses

**Figure 1:**
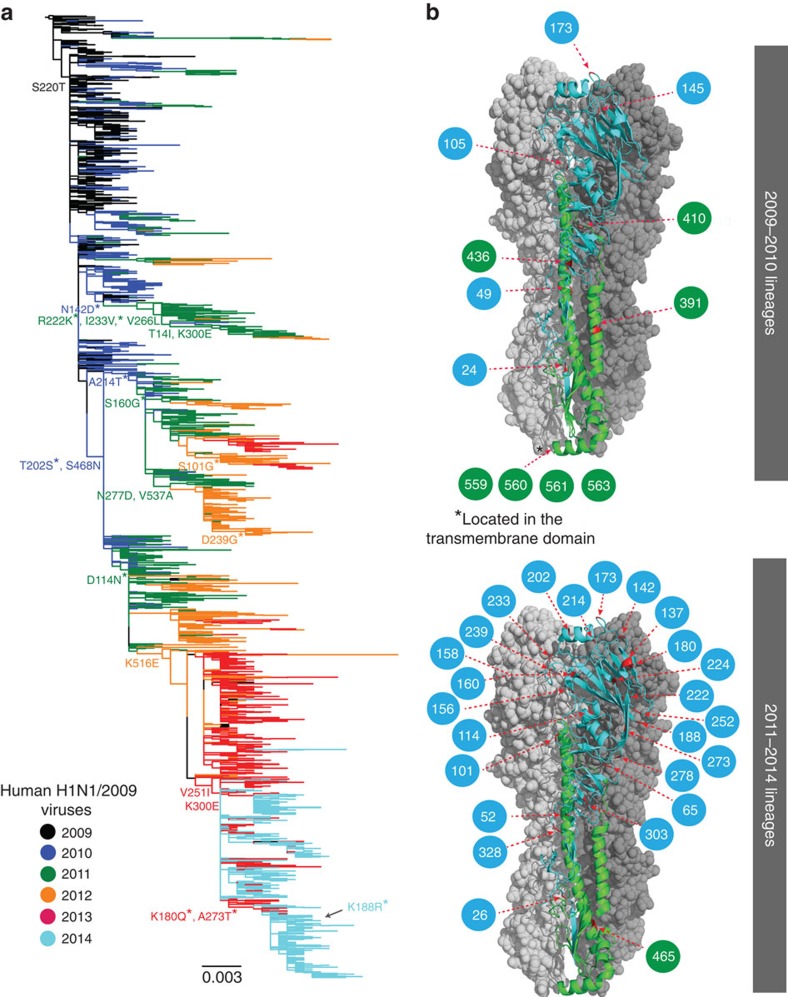
Evolution of human H1N1/2009 viruses and structural mapping of positively selected sites on the HA molecule. (**a**) Maximum likelihood phylogeny of 2,280 human H1-HA sequences from 2009 to 2014 with branches coloured by year of virus isolation. Representative amino-acid substitutions are mapped at the major tree nodes. Sites marked with asterisks (*) indicate positively selected sites identified by MEME method, with the significance level at *P*<0.05. Scale bar represents number of substitutions per site. (**b**) Mapping of positively selected amino-acid sites identified by MEME method onto the three-dimensional structure of the HA glycoprotein of H1N1/2009 virus (Protein Data Bank code: 3LZG) for the pandemic (2009–2010) and post-pandemic (2011–2014) periods. The monomer shows the HA1 subunit in light blue and the HA2 subunit in green. The numbers in coloured circles denote codon alignment number and their locations in the three-dimensional structure are indicated with red arrows.

Analysis of all H1-HA influenza viruses shows that H1N1/2009 viruses (Supplementary Fig. 1, red branches) completely replaced and segregated from the previous seasonal H1N1 viruses that circulated in humans before the 2009 pandemic (Supplementary Fig. 1, blue branches). The global phylogeny of the *HA* gene of H1N1/2009 viruses revealed a comb-like appearance during the early phase of the pandemic in 2009–2010 (Fig. 1a). This pattern is indicative of a rapid increase in genetic diversity in the absence of strong selective pressures with virus spread determined by stochastic events and rapid transmission, as would be expected of a virus population infecting a predominantly naive human population. In contrast, post-pandemic H1N1/2009 viruses isolated since 2011 have exhibited a ladder-like phylogeny, characteristic of viruses subject to continuous antigenic drift, typical of human seasonal influenza viruses (Fig. 1a, Supplementary Fig. 2). This change in evolutionary pattern coincided with the emergence of two distinct H1N1/2009 lineages from 2011 onwards, although one of these lineages went extinct, resulting in circulation of a single dominant H1N1/2009 lineage in 2014 (Fig. 1a).

We also mapped the amino-acid substitutions occurring at the nodes of H1N1/2009 HA phylogeny using maximum likelihood^36^. This revealed a number of amino-acid changes along the backbone of the HA phylogeny, particularly after 2011, possibly indicating directional selection acting on the H1N1 HA following this period (Fig. 1a). Selection analyses on the combined 2009–2014 H1N1/2009 data showed that the *HA*, *NA*, *M2* and *NS* genes had a higher global d_N_/d_S_ ratios than all other genes and identified two amino-acid sites in the HA under significant positive selection (Supplementary Table 1). These sites were situated either at antigenic sites or in proximity to the receptor-binding pocket, namely Q180K (Sa antigenic site) and D239G (Ca2 antigenic site)^37^. Taken together, these results suggest that by 2011, a critical population size had acquired immunity to H1N1/2009 virus either through natural infection or ongoing vaccinations and that antibody-mediated selection may have started to drive virus evolution.

**Figure 2:**
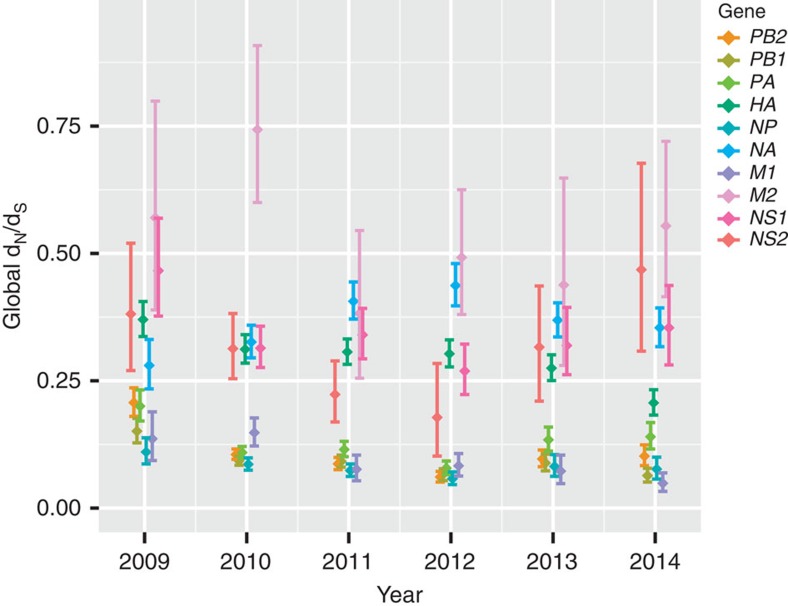
Selection pressures on human H1N1/2009 viruses over time. Yearly estimates of global d_N_/d_S_ ratios for each gene using SLAC method, with the significance level at *P*<0.05. Each gene data set per epidemic year consisted of up to 500 randomly selected global sequences. The means of d_N_/d_S_ ratios are indicated by diamond-shaped symbols with error bars representing 95% confidence intervals. Coloured bars correspond to different genes in the following order: polymerase basic 2 (*PB2*), polymerase basic 1 (*PB1*), polymerase acidic (*PA*), haemagglutinin (*HA*), nucleoprotein (*NP*), neuraminidase (*NA*), two matrix proteins (*M1* and *M2*), and two non-structural proteins (*NS1* and *NS2*).

To investigate this apparent transition further, we used the single likelihood ancestor counting (SLAC) method^38^ to compare the selection pressures acting on the H1N1/2009 virus genes during the early (2009–2010) and post-pandemic (2011–2014) periods (Table 1). Global d_N_/d_S_ estimates were generally higher during the pandemic than those observed in the post-pandemic period (Table 1). In addition, yearly estimates of global d_N_/d_S_ values for each gene segment were generally elevated in 2009 compared with subsequent years, with the exception of the *NA* gene (Fig. 2, Supplementary Table 2). The elevated d_N_/d_S_ values observed in the pandemic period could have resulted from relaxed selection following interspecies transmission^39^, or may be indicative of adaptation to the new human host. Conversely, an increase in d_N_/d_S_ value is apparent in the *NA* gene until 2012 that we postulate may be due to the observed imbalance in HA and NA functionality in some H1N1/2009 viruses^40^ or broad NA-related immunity^41^.

The rate ratio of internal d_N_/d_S_ versus external d_N_/d_S_ branches was also relatively higher in most genes during the pandemic period compared to the post-pandemic period (Table 1). The lower internal/external d_N_/d_S_ ratio observed in the post-pandemic period may indicate an increase in the deleterious amino-acid mutations that were eventually removed from the population due to competition, consistent with observations made for seasonal influenza A viruses^42^.

To determine whether individual sites were under positive or relaxed selection, we compared the results of the M8 model^43^ and the mixed effects model of evolution (MEME) method^44^. Owing to the restricted rate variation of synonymous sites in the M8 method, relaxed selection constraints are often reported as positive selection. Whereas MEME, which takes synonymous rate variation into account, detects residues that are under both pervasive positive selection and episodic selection with an increase in power than the M8 model alone^38^. By comparing the two sets of results we can determine the respective contribution of relaxed selective constraints and sites purely under positive selection. Our results show a greater number of sites in the *PB2*, *PA*, *HA*, *NP* and *M2* genes were likely under relaxed selection in the pandemic than in the post-pandemic period (Table 1). The other genes either showed no change or an increase in the sites under relaxed selection.

The results of the MEME analyses deduced 12 versus 25 positively selected amino-acid residues in the HA for the pandemic and post-pandemic viruses (Table 1). However, we observed marked differences in the positions of those mutations in the HA (Fig. 1b) that were statistically significant (at a *P* value of 0.0003): 4 sites (pandemic) versus 24 sites (post-pandemic) in the *HA1* gene, and 7 sites (pandemic) versus 1 site (post-pandemic) in the *HA2* gene. For viruses from the pandemic period, residues under positive selection were mostly located on the HA stem region that is generally conserved (Fig. 1b), consistent with the observation that early pandemic lineages were subject to selective pressures favouring adaptation to the new host. Alternatively, this could be at least partially immunologically driven as the HA stalk is more likely to contain conserved epitopes shared with other HA subtypes such as seasonal H3N2 and H1N1 (ref. 45). However, two of those mutations are located internally while one externally exposed amino acid is not known to be antigenic. Comparatively, selection pressure in post-pandemic viruses involved an increase in amino-acid mutations in the HA globular head (Fig. 1b), leading to the increased diversifying selection in 2011–2014 lineages most likely in response to host immune pressure. Notably, substitutions at some of the sites under positive selection, including residues 224 and those located in the 150 loop, are known to cause antigenic change and enhanced replication efficiency of H1N1 viruses^46^,^47^.

Results of the selection analysis on the remaining seven gene segments were consistent with this hypothesis (Table 1). For example, during the pandemic period there was stronger positive selection acting on the polymerase and nucleoprotein (*NP*) genes that was reduced in the post-pandemic period. In summary, we found evidence of a relaxation in selection constraints that decreased throughout the study period. However, our results also show that positive selection in the early pandemic period appears to have been predominantly driven by adaptation to the new human host, while in the later post-pandemic period positive selection was directed towards the viruses escaping the host immune response.

### Antigenic evolution of H1N1/2009

**Figure 3:**
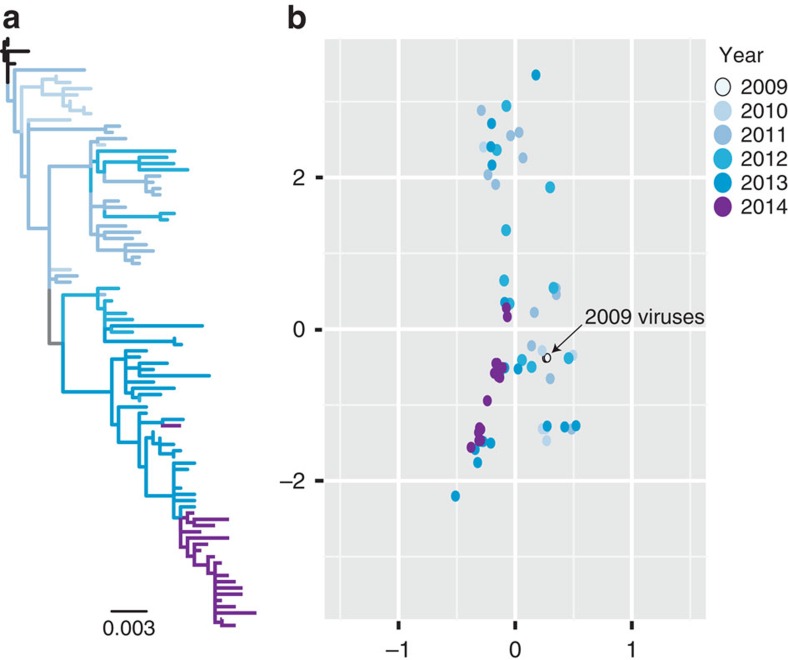
Antigenic evolution of human H1N1/2009 viruses. (**a**) Maximum likelihood phylogeny of the *HA* gene of Australian viruses used in the HI assay. Coloured branches represent the year of virus isolation on the right. Scale bar represents number of substitutions per site. (**b**) Antigenic cartography of H1N1/2009 viruses reconstructed based on HI assays of pandemic and post-pandemic strains against a panel of polyclonal antisera. Coloured dots correspond to the year of virus isolation. The position of viruses isolated in 2009 is indicated by the black arrow.

To further understand the effect of the observed amino-acid changes on the antigenicity of these viruses, we conducted HA inhibition (HI) assays of 66 representative H1N1/2009 viruses collected in Australia (Supplementary Fig. 2) from 2009 to 2014 using a panel of ferret polyclonal antisera (Supplementary Table 3). Antigenic cartography of HI titres revealed little antigenic drift in the H1N1/2009 viruses (Fig. 3), consistent with the California-like viruses being recommended as either monovalent or part of the trivalent/quadrivalent influenza vaccine component by the WHO from 2009 to 2016. However, the viruses from the pandemic period and the early post-pandemic period showed a broader antigenic diversity in comparison to viruses from the late post-pandemic period, particularly the 2014 strains. The broad antigenic diversity observed for the earlier viruses (Fig. 3) corresponds to a period of higher relaxed selection but where there is evidence that adaptive selection was targeted at the stalk of the HA (Fig. 1b). For the later viruses, we observed a more restricted antigenic diversity (Fig. 3) that may have risen when selection was more directed to escape the host immune response (Fig. 1b). Despite these changes in antigenic diversity, it must be noted that the 2014 viruses remain antigenically and phenotypically similar to the 2009 strains, and no update of the vaccine strain (an A/California/7/2009-like virus) has been recommended by WHO to date.

### Population dynamics of the H1N1/2009 virus

Phylogenetic analysis of the *HA* gene sequences showed no significant difference in the TMRCAs (time to the most recent common ancestors) of H1N1/2009 viruses from Mexico and the United States, but their TMRCAs were significantly earlier (Bayes factor (BF)>7) than all other regions tested (Supplementary Table 4). This suggests that the H1N1/2009 virus may have circulated undetected in North America before the pandemic was detected.

**Figure 4:**
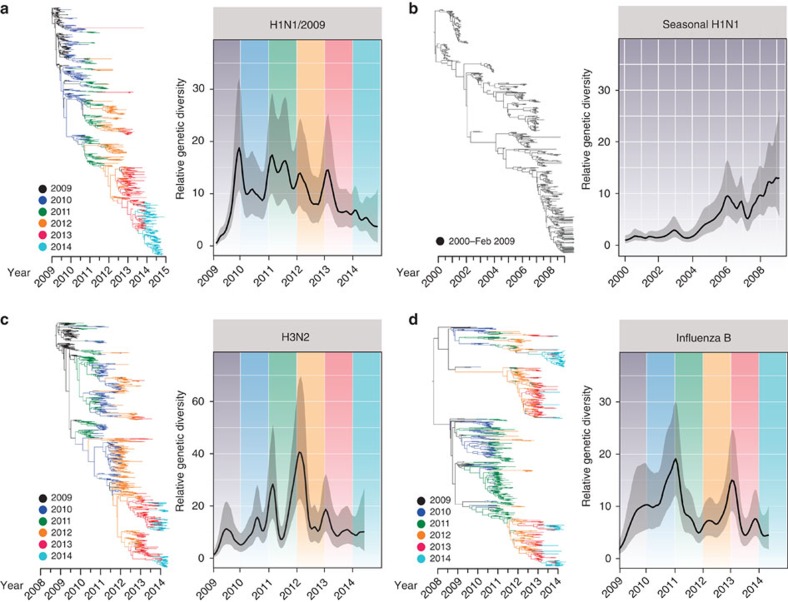
Temporal phylogenies and epidemic patterns of human influenza viruses. Evolution of the *HA* genes of (**a**) H1N1/2009 viruses (2009–2014), (**b**) seasonal H1N1 viruses (2000–February 2009), (**c**) H3N2 viruses (2009–2014) and (**d**) influenza B viruses (2009–2014). Phylogenies were inferred using the uncorrelated lognormal relaxed clock model with branches coloured by year of virus isolation and relative genetic diversity estimated using the Gaussian Markov Random Field (GMRF) model. Solid black lines in the GMRF plot represent mean relative genetic diversity while the corresponding grey shades indicate the 95% HPD intervals.

Coalescent reconstruction of the global outbreak revealed oscillating patterns in relative genetic diversity of H1N1/2009 viruses after an initial rapid increase during the early pandemic spread that broadly corresponded to the Northern and Southern Hemisphere winters (Fig. 4a). These results indicate that after emergence, the H1N1/2009 viruses rapidly adapted to form seasonal epidemic patterns with the temperate regions typically exhibiting rapid increase in relative genetic diversity followed by distinct seasonal bottlenecks. However, these patterns did show variation in seasonal intensity with smaller epidemics observed in mid-2010 and mid-2013, and virus diversity was markedly reduced throughout 2014. In contrast, the global seasonal H1N1 lineages (Fig. 4b) showed weaker seasonal fluctuations over time due to the co-circulation of multiple virus lineages for longer periods of time. Comparison with the population dynamics of human H3N2 and influenza B viruses indicates that these smaller epidemics of H1N1/2009 correlated with higher incidence and increased relative genetic diversity of H3N2 (Fig. 4c) and influenza B (Fig. 4d). Interestingly, the overall relative genetic diversity of H1N1/2009 viruses was comparatively lower than both influenza B and H3N2 viruses. If we assume the behaviour of all influenza is similar regardless of subtype, then the observed differences may be interpreted as absolute differences in seasonal epidemic size. While the validity of this assumption is debatable, it does appear to correlate with lower levels of H1N1/2009 incidence observed in mid-2010, mid-2013 and 2014.

**Figure 5:**
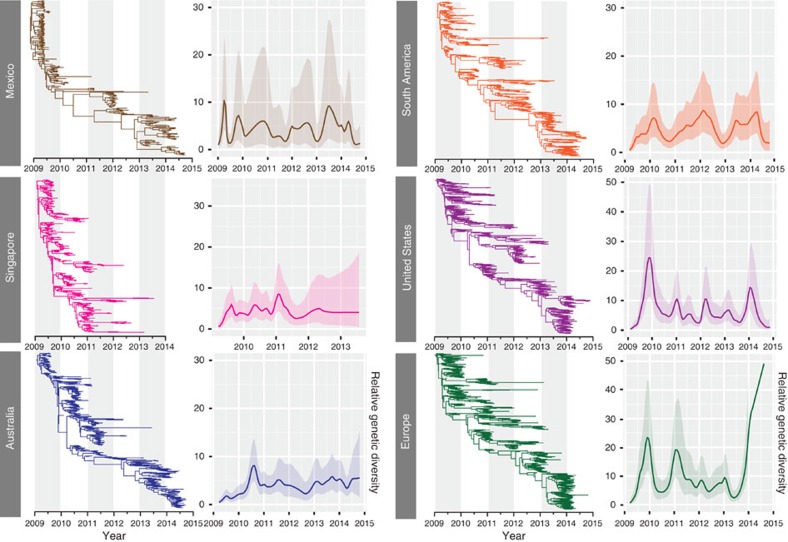
Comparative phylogenetic analyses and population dynamics of H1-HA viruses circulating in different geographical regions, 2009–2014. Phylogenies were inferred using the uncorrelated lognormal relaxed clock model and relative genetic diversity estimated using the Gaussian Markov Random Field (GMRF) model. Solid lines in the GMRF plot represent the mean relative genetic diversity through time, while the corresponding shaded areas indicate the 95% HPD intervals.

We compared the relative genetic diversity of 12 different geographic regions with coinciding epidemic peaks, representing the tropics and the Northern and Southern Hemispheres (Fig. 5 and Supplementary Fig. 3). Our results showed that Mexico exhibited the earliest peak during 2009 (Fig. 5). However, despite the earliest H1N1 reports from the United States starting in April 2009 (ref. 5), the relative genetic diversity peaked in early December 2009, suggesting that the initial outbreak was limited in spread and duration. Following the introduction of the H1N1/2009 virus, the studied locations exhibited patterns typical of human seasonal influenza. For example, Europe and the United States showed patterns of increase over the winter months, while tropical locations typically showed lower levels of relative genetic diversity and biannual epidemic peaks that corresponded to the seasonal outbreaks of the Northern and Southern Hemispheres (Fig. 5 and Supplementary Fig. 3). There were exceptions where seasonal patterns took longer to become established, such as in Japan and other regions that showed little or no H1N1/2009 activity in some years, that most likely corresponded to local epidemics being dominated by H3N2 and influenza B viruses.

We estimated the basic reproductive ratio (*R*_0_)—the average number of secondary infections arising from primary infections^19^—during the exponential growth phase of each epidemic season (see Fig. 5) for 5 of the 12 regions described above, representing the tropics (Singapore), the Northern (Mexico, Europe and the United States) and Southern Hemispheres (Australia). Our findings showed that *R*_0_ was slightly higher during first pandemic peak (mean *R*_0_=1.05–1.10) with a slightly lower estimate for subsequent years (mean *R*_0_=1.00–1.03 in 2010–2014) in the tropics and in the Northern Hemisphere (Supplementary Table 5). Although there was a slight increase in *R*_0_ estimated in Australia, this change was not significant (mean *R*_0_=1.00, 95% highest posterior density (HPD)=0.99–1.03 versus mean *R*_0_=1.03, 95% HPD=1.00–1.11).

### Spatial dynamics of H1N1/2009 viruses

**Figure 6:**
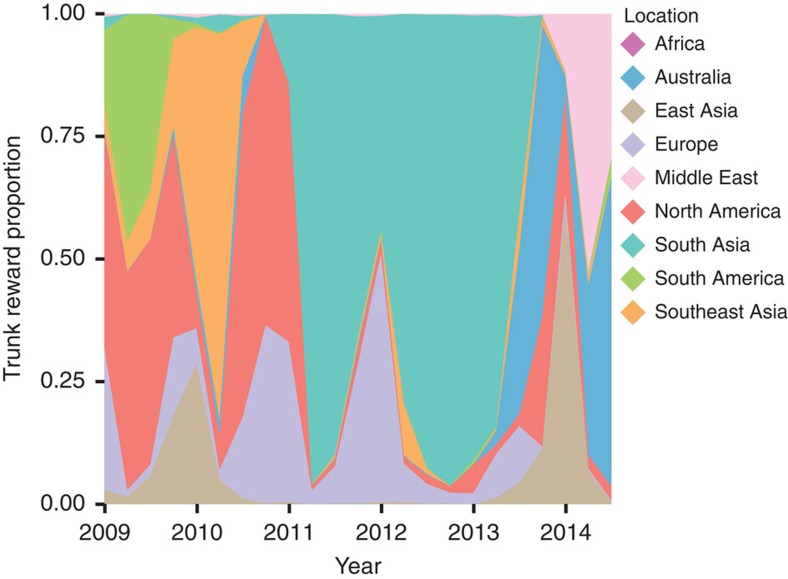
Proportional ancestral location states on the phylogenetic tree trunk estimated for each location over time. The waiting time between geographic location transitions was inferred using the continuous-time Markov chain model. The trunk reward proportion for each geographic location from 2009 through 2014 was determined from an analysis of 2,225 HA sequences of H1N1/2009 viruses. Shaded areas represent the trunk proportions over time for the nine geographical locations included in the analysis.

To understand the global circulation of H1N1/2009 viruses, we reconstructed the past spatial transmission patterns for each annual global epidemic period, from October to September each year, inferred from estimates of genetic diversity (Fig. 4a) for nine geographic regions (that is, Africa, Australia, East Asia, Europe, the Middle East, North America, South America, South Asia and Southeast Asia). We also estimated the reward times for the proportional trunk locations^48^ during 2009–2014 (Fig. 6). Our analysis shows that annual epidemics emerge from a globally migrating population where the ancestral population occupies multiple locations on the phylogenetic tree trunk. For example, Southeast Asia and North America occupied the trunk in early and late 2010, respectively, while South Asia had the strongest signal of any region with >50% support from early 2011 to mid-2013, after which Australia was identified as the dominant trunk location (Fig. 6, Supplementary Table 6).

**Figure 7:**
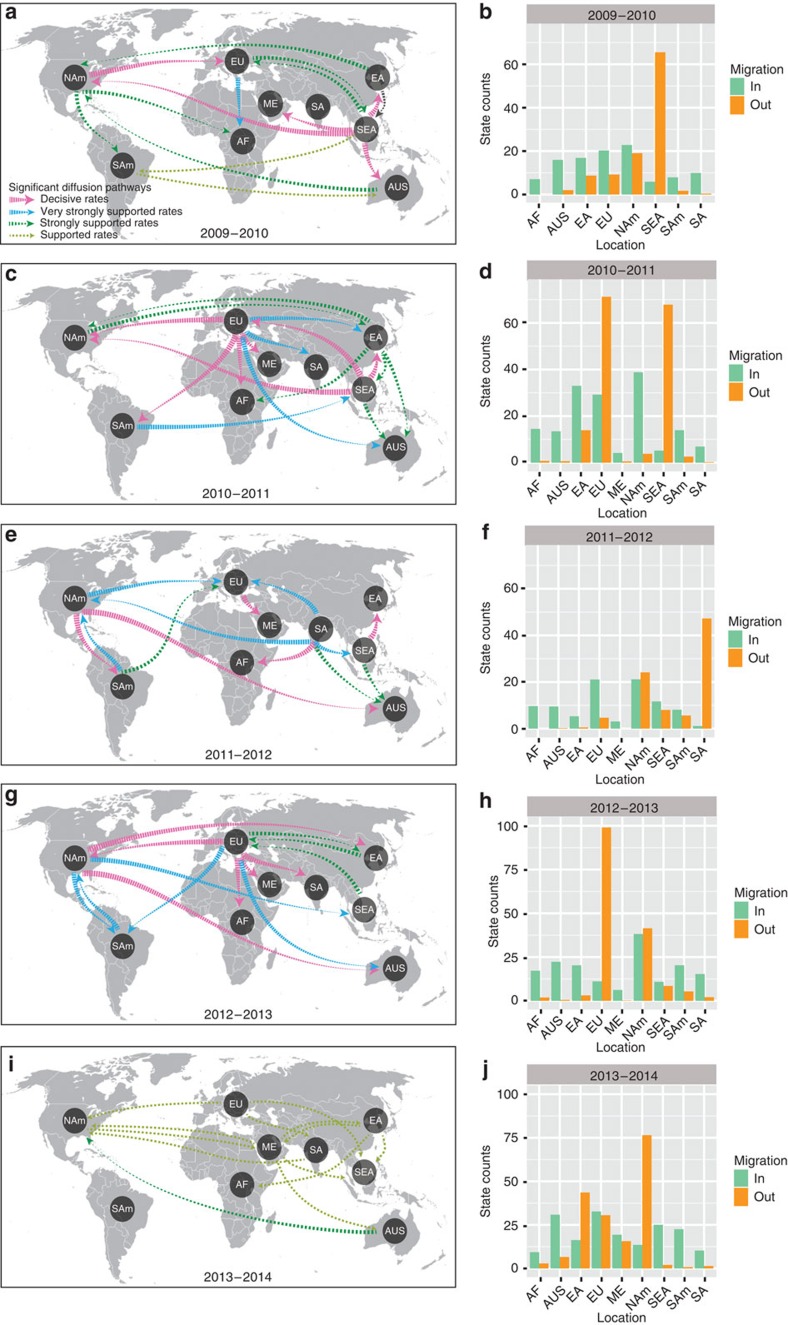
Spatial diffusion of H1N1/2009 viruses. Spatial diffusion pathways and histograms of total number of state transitions for (**a**,**b**) 2009–2010, (**c**,**d**) 2010–2011, (**e**,**f**) 2011–2012, (**g**,**h**) 2012–2013 and (**i**,**j**) 2013–2014. Global epidemic year was defined as October to the following September based on observed epidemics in Fig. 3a. Significant epidemiological unidirectional pathways from one location to another are indicated on the maps. Thickness of coloured lines represents statistically supported migration rates with a mean indicator of >0.5: pink arrows, decisive rates with BF≥1,000; blue arrows, very strongly supported rates with 100≤BF<1,000; dark green arrows, strongly supported rates with 10≤BF<100; and light green arrows, supported rates with 3≤BF<10. AF, Africa; AUS, Australia; EA, East Asia; EU, Europe; NAm, North America; SEA, Southeast Asia; SAm, South America; SA, South Asia.

In 2009–2010, our phylogeographic analysis indicate five significant migration links with mean rates from 1.24 to 2.24 (decisive support with BF ≥1,000), with four originating from Southeast Asia to East Asia, Australia, the Middle East and North America and a fifth from North America to Europe (pink arrows in Fig. 7a; Supplementary Table 7). Very strongly and strongly supported diffusion rates were also indicated between numerous regions worldwide. In total, resulting diffusion rates indicate that in 2010 Southeast Asia played a key role in seeding the H1N1/2009 virus epidemics. This is further supported by the number of observed state changes (that is, the number of geographical state transition/year) with migration from Southeast Asia being much greater than any other location included in our analysis (Fig. 7b, Supplementary Table 8). We also plotted the total mean rate per MCMC (Markov Chain Monte Carlo) jump for Southeast Asia, which clearly demonstrated an outward bias in total mean rates from Southeast Asia to all other locations in 2010, resulting in an asymmetrical plot (Supplementary Fig. 4).

Seven migration pathways with decisive support were recognized in 2010–2011 with four from Europe to Africa, Middle East, North America and South America and the other three from Southeast Asia to East Asia, Europe and to North America (Fig. 7c). High mean actual rates were observed for migration from Southeast Asia to Europe and from Europe to North America (2.93 and 3.09, respectively; Supplementary Table 7). State change counts reflected this dynamic with outward migration from Europe and Southeast Asia dominating (Fig. 7d). However, total mean rates showed migration out of Southeast Asia to all other locations but equal numbers of inward and outward mean rates for Europe (Supplementary Fig. 5). As such our analysis indicates that in 2011 Southeast Asia acted as a primary seeding population while Europe established strong epidemiological links with multiple regions in this transmission network.

Influenza migration patterns in 2011–2012 showed a marked contrast with previous years, with at least five strong epidemiological links originating from South Asia to other locations (Fig. 7e, Supplementary Table 7) and significant pathways (BF≥1,000) reported from North America to Australia and South America, Europe to the Middle East and Southeast Asia to East Asia. While multiple geographical locations therefore seem to act as seeding populations in this epidemic period, state counts and total mean rates support the idea that South Asia may have acted as a seeding population (Fig. 7f and Supplementary Fig. 6) that is concordant with the highest trunk proportion being located in South Asia in 2011–2012 (Fig. 6).

In 2013, seven strongly supported pathways (BF>10) from Europe were apparent, generally indicating migration from the Northern Hemisphere to Asia, the Tropics and the Southern Hemisphere (Fig. 7g, Supplementary Table 7). Results indicated that North America also established statistically strong migration links to Australia, East Asia, South America and Southeast Asia. However, state counts and total mean rates suggest that, while Europe acted as the potential seeding population and North America showed more or less equal numbers of outward and inward migrations, there was a slight outward bias in both state changes and total mean rates in the North American viruses (Fig. 7h and Supplementary Fig. 7). Interestingly, extreme weather events in 2013 led to an unusually cold European and North American winter and spring^49^ that may have favoured increased influenza activity^50^ and could explain the observed virus migration patterns.

The 2014 epidemic year was comparatively less distinctive and rather indistinguishable in revealing migration patterns (Fig. 7i, Supplementary Table 7) as the diffusion rates had less statistical support than previous years. This also matches the trunk reward times for 2014 where multiple ancestral populations were identified (Fig. 6). The state counts suggest a migration out of North America is probable (Fig. 7j), although the total mean rates indicate higher rates from Europe to South Asia and Middle East to North America (Supplementary Table 7).

### Reassortment of H1N1/2009 viruses

Temporal phylogenies of global genomic sequences indicate that the mean substitution rates of H1N1/2009 *HA* and *NA* genes were 5.34 × 10^−3^ (95% HPD: 4.76–5.95 × 10^−3^) and 5.21 × 10^−3^ (95% HPD: 4.64–5.82 × 10^−3^) subs per site per year, respectively, which were higher than the mean rates observed for the internal genes (3.52–5.34 × 10^−3^ subs per site per year; Table 2). These rates were generally higher than those inferred during the pandemic period^3^,^4^. In comparison, the seasonal H1N1 (2000–February 2009) had a significantly slower HA evolutionary rate than the H1N1/2009 viruses, with a mean and HPD values of 4.37 × 10^−3^ subs per site per year (3.88–4.85 × 10^−3^ subs per site per year, BF=1,488). Correspondingly, mean root heights of each gene segment of the H1N1/2009 viruses varied from December 2008 to January 2009, which dated approximately four months earlier before the outbreak (Supplementary Figs 8–10). These results were largely consistent with estimates obtained during the early pandemic period^3^.

**Figure 8:**
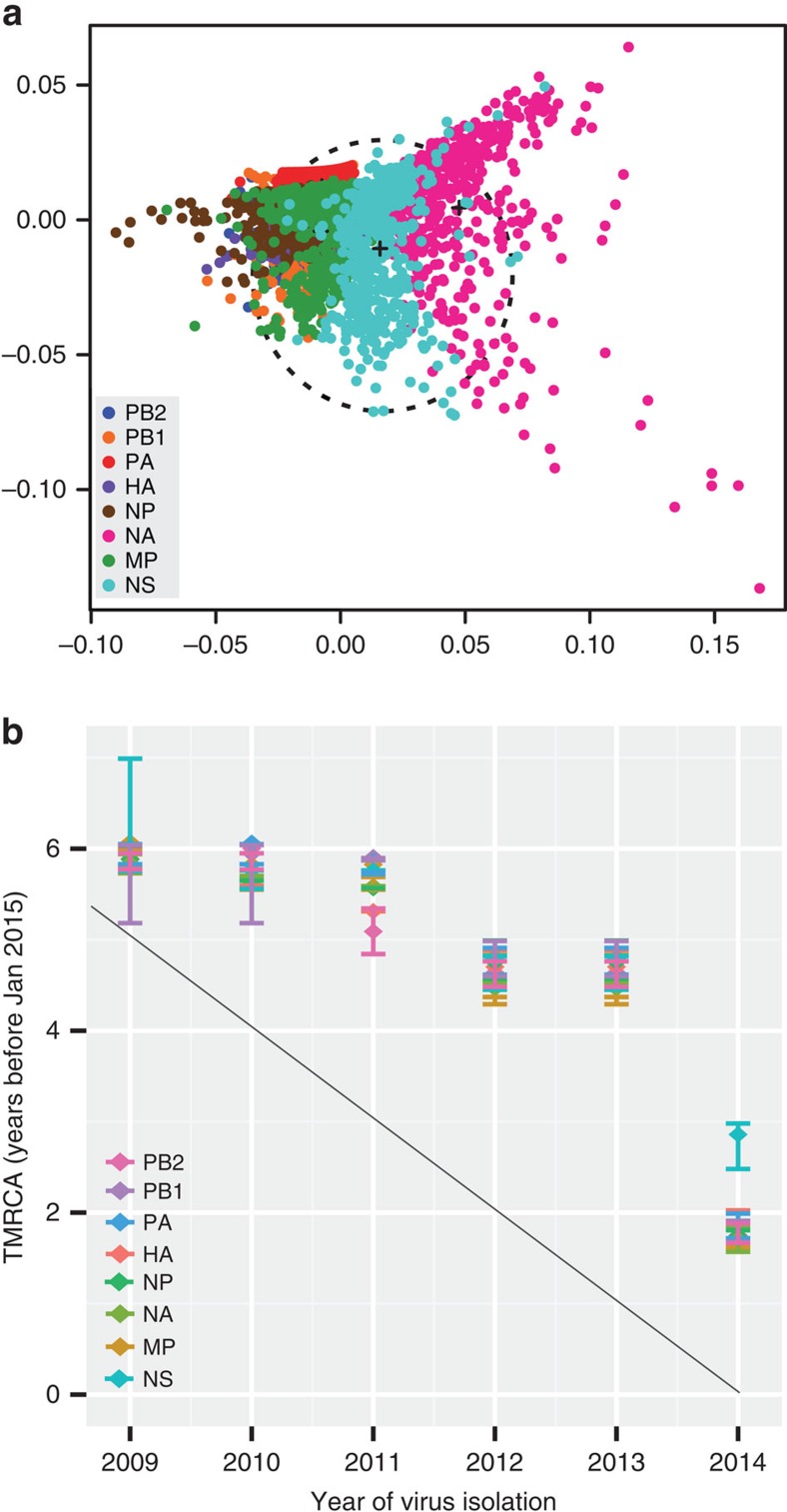
Genomic reassortment and global genetic diversity of H1N1/2009 viruses. (**a**) Multidimensional scaling plot of uncertainty of TMRCA between samples of 500 trees for each segment of pandemic H1N1/2009 viruses sampled between 2009 and 2014. The space occupied by human H3N2 viruses^51^ is indicated by the dashed circle. (**b**) The TMRCA of each genomic segment of H1N1/2009 viruses circulating in each year. The TMRCAs of all segments in 2014 were dated more recently than 2009–2013 lineages, indicative of reduced genetic diversity across the whole genome. Values shown indicate the mean TMRCAs and the 95% HPD intervals from the Bayesian MCMC analysis. The diagonal line is traced along January of each year.

To characterize the genomic reassortment of the H1N1/2009 viruses, we utilized a multidimensional scaling of the TMRCAs of each viral segment that were calculated from sets of 500 BEAST trees^51^. In Fig. 8a, the spread of the same coloured gene clouds represents uncertainty in phylogenetic history of a gene, while the overlapping of the gene clouds for each gene observed in our analysis indicate homogenous phylogenetic histories and that genomic reassortment of H1N1/2009 viruses has to date been rare.

Analysis of the mean TMRCAs and 95% HPDs for all gene segments based on each epidemic year showed that TMRCAs for viruses isolated in 2009–2013 fell progressively further before the start of the epidemic season (Fig. 8b), demonstrating that there was diversification of H1N1/2009 as it persisted in humans and also indicating that this diversity persisted across multiple epidemics, in a pattern typical of seasonal influenza viruses^51^. Notably, in 2014 the TMRCAs of all genes except the *NS* fell very close to the start of the year, indicative of a global reduction in virus genetic diversity circulating in humans (Fig. 8b). The TMRCA of the *NS* gene was older than the TMRCAs of all other genes (Supplementary Table 9), due to the circulation of two NS populations from different locations, mainly Europe and North America. Mapping of the amino-acid substitutions at the nodes of H1N1/2009 phylogenies indicates that this reduction in genetic diversity is associated with mutations in the HA (V251I and K300E), NA (I34V, I321V and K432E) and PA (K361R and R362K) (Fig. 1a and Supplementary Fig. 9). The two HA mutations are located in diversified B-cell/antibody epitopes^52^ while no functional role has been identified experimentally for the NA or PA mutations.

## Discussion

Our study provides a comprehensive investigation of the evolutionary dynamics of H1N1/2009 virus as it has adapted to its new human host. We demonstrate that the early pandemic lineages exhibited markedly higher global d_N_/d_S_ rates due to relaxed selective constraints directly following zoonotic transmission, in combination with different gene mutations that evolved to adapt to the new host. In particular, during the pandemic phase, mutations primarily accumulated in the stalk of the HA and in the polymerase genes, and mutations in these genes have been associated in humans with increased polymerase activity and replication and with changes in host specificity^53^,^54^,^55^. Interestingly, mutations in the HA stalk are associated with increased HA protein stability and are critical in the airborne transmission of avian viruses in mammals^56^. While it is likely that H1N1/2009 viruses were, at least to some degree, pre-adapted to humans during circulation in swine, the predominance of amino-acid mutations in the HA stalk and replicative machinery is indicative of host adaptation. These amino-acid residues therefore warrant further investigation as potential targets for therapeutic intervention.

In contrast, in the post-pandemic period (2011 onwards) there was a general decrease in global d_N_/d_S_ rates and relaxed selection in most genes. We also observed a reduction of positively selected sites in the polymerase genes but a greater number of mutations occurring in the HA globular head, similar to mutational patterns seen in seasonal influenza viruses^57^,^58^. This change in the nature of the selective pressures acting on the HA corroborates the results of the antigenic assays where we observed an initial expansion in antigenic diversity followed by restricted antigenic diversity in 2014 viruses that also coincides with the observed reduction in virus genetic diversity in 2014. These changes are most likely driven by increased anti-H1N1/2009 antibodies in humans that in turn has led to directed selection in the H1N1/2009 population.

Although the A/California/07/2009-like virus is still the WHO-recommended vaccine strain, this is likely because immune-driven selection was not observed until 2 years after the pandemic and we have only just started to observe related changes in antigenic evolution. Our observations are also consistent with a recent study that used human antiserum from individuals vaccinated against A/California/07/2009, and where they detected 8 out of 10 human antisera that demonstrated reduced reactivity against 2014 H1N1/2009 isolates^52^.

Reconstruction of H1N1/2009 virus time to coalesce from temporal phylogenies showed that different geographical regions generated local epidemic peaks that matched with their respective annual winter seasons, followed by seasonal genetic bottlenecks. Furthermore, inferred spatial dynamics identified multiple ancestral populations and indicated that multiple geographical regions might act as a potential seed for local epidemics. Human seasonal H1N1 and H3N2 influenza viruses have been previously shown to undergo global selective sweeps^51^, allowing the extinction of existing strains and leading to replacement with new strains. Even though such behaviour has not been observed for the H1N1/2009 virus, our study provides a detailed description of the early evolutionary dynamics of this lineage highlighting contrasting evolutionary patterns to the previously circulating seasonal H1N1 and co-circulating H3N2 virus lineages. Continued genomic and antigenic investigations of the H1N1/2009 viruses are clearly required to determine their long-term population dynamics.

## Methods

### Virus surveillance, isolation and sequencing

Influenza surveillance was conducted for 5 years in Singapore during June 2009–July 2013 and 1,310 nasopharyngeal swabs were collected from patients who presented with signs of flu-like symptoms at National University Hospital and at Singapore General Hospital. Of these, 613 samples were selected based on collection date, cultured in Madin–Darby canine kidney SIAT-1 cells and then screened for H1N1/2009 viruses using PCR with reverse transcription, which found a total of 305 of 613 (49.7%) positives. From these we then obtained 214 full genomes of human H1N1/2009 viruses. All viral sequences were assembled and edited using Geneious Pro 7.1 (Biomatters Ltd).

### Phylogenetic analysis

A H1 phylogeny was generated using a data set containing 5,804 HA sequences from different hosts (that is, avian, swine and human) and collected between 1930 and 2014 was reconstructed using maximum likelihood analyses using the general time reversible+Γ nucleotide substitution model in RAxML v.8.0.14 (ref. 59) (Supplementary Fig. 1). All data were downloaded from the NCBI Influenza Virus Resource and GISAID EpiFlu database. Analysis of only human H1N1/2009 H1-HA (2009–2014) was then conducted with over 6,000 globally sampled H1N1/2009 sequences of viruses isolated from Africa, Australia, East Asia, Europe, the Middle East, North America, South America, South Asia and Southeast Asia, in addition to the 214 genomes from this study. From this, we randomly sampled 380 H1-HA sequences per year to give a final data set of 2,280 H1-HA sequences and inferred a maximum likelihood phylogeny as described above (Fig. 1 and Supplementary Fig. 2a). For genomic analyses (see details below), we also generated data sets of 485 sequences for all eight gene segments by randomly sampling ∼80 sequences per year. Ancestral codon substitutions for each gene were estimated using baseml, as implemented in PAML, using ML trees inferred in RAxML as described above. Ancestral substitutions were then transcribed onto the gene phylogenies (Fig. 1, Supplementary Figs 8 and 9) using the treesub programme^38^.

### Natural selection

To compare the selection pressures acting on H1N1/2009 viruses in the pandemic (2009–2010) and post-pandemic (2011–2014) periods and across all years (2009–2014), globally representative data sets were randomly sampled for each of the 10 main influenza A proteins (PB2, PB1, PA, HA, NP, NA, M1, M2, NS1 and NS2). Global non-synonymous to synonymous (d_N_/d_S_) rate ratios were estimated with the SLAC method^38^ in Datamonkey^60^ and we compared the branch-wise dN/dS ratios of the internal and external branches using the two-model ratio in CODEML available in PAML^61^. The MEME method in Datamonkey and the Bayes empirical Bayes (M8 model+BEB) method^43^ in PAML were then used to identify the positively selected sites for each segment, with the significance level at *P*<0.05. A year-by-year comparison of global d_N_/d_S_ was also calculated using SLAC. To visualize the locations of positively selected sites identified by MEME for the pandemic and post-pandemic periods, each identified amino-acid residue was mapped onto a three-dimensional structure of the H1-HA of H1N1/2009 virus (Protein Data Bank code: 3LZG) using PyMOL (Molecular Graphics System, version 1.5.0.4 Schrödinger, LLC).

### Antigenic cartography

A maximum likelihood tree was reconstructed using 95 H1-HA sequences of the H1N1/2009 viruses isolated in Australia (Fig. 3a and Supplementary Fig. 2c). To visualize the phylogenetic positions of these viruses they were highlighted in the large global phylogeny (red branches in Supplementary Fig. 2). A total of 66 representative viruses were subsequently selected and included in HI assays. The HI assays were conducted to measure the cross-reactivity of selected H1N1/2009 viruses (collected from 2009 to 2014) using a panel of post-infection ferret sera raised against a range of H1N1/2009 viruses (Supplementary Table 3). The resulting HI titres represent the reciprocal of the last dilution that inhibits the ability of the antibody to inhibit the virus from agglutinating turkey red blood cells. To visualize the antigenic evolution, an antigenic cartography was constructed using the two-dimensional coordinates generated by Antigen Map v.1.0 (ref. 62).

### Temporal dynamics of human influenza subtypes

To compare the evolutionary dynamics of H1N1/2009 with other human influenza subtypes, global HA data sets were downloaded from GenBank and GISAID for H1N1/2009 (895 sequences), A/H3N2 (845 sequences) and influenza B (839 sequences) covering the period from April 2009 to May 2014. The H1-HA sequences of previous seasonal H1N1 viruses (508 sequences) from 2000–February 2009 were also analysed. The coalescent-based Gaussian Markov random field (GMRF) method with the time-aware smoothing parameter^63^ was used for reconstructing past population dynamics as implemented in BEAST v.1.8.1 (ref. 64). For all analyses, we used the uncorrelated lognormal relaxed molecular clock, the SRD06 codon position model^65^, to allow partitioning for codon positions (1+2 positions and 3 position), with the HKY85+Γ substitution model applied to these codon divisions. Four independent analyses of 100 million generations and with sampling every 10,000 generations were performed. Convergence of runs was checked in Tracer v.1.6 (available from http://beast.bio.ed.ac.uk/Tracer) to ensure adequate mixing and that all parameters reached adequate effective sample size and runs were combined. The GMRF plots and the maximum clade credibility trees were visualized in FigTree v1.4.2 (available from http://tree.bio.ed.ac.uk/software/figtree).

### Regional GMRF skyride analyses of H1N1/2009 virus

We compared the evolutionary dynamics of H1N1/2009 viruses using the H1-HA sequence data from viruses isolated from 12 representative geographical regions based on data availability during 2009–2014: Australia (511 sequences), Brazil (239 sequences), China (249 sequences), Europe (629 sequences), Hong Kong (225 sequences), India (194 sequences), Japan (641 sequences), Mexico (239 sequences), Russia (200 sequences), Singapore (348 sequences), South America (489 sequences) and the United States (630 sequences). For each regional data set, the GMRF skyride plots and the maximum clade credibility trees were reconstructed in BEAST as described above. A BF test was used to calculate whether there were significant differences for the TMRCAs of different regions.

### Estimation of reproductive ratio (*R*_0_)

Five geographical localities (Australia, Europe, Singapore, Mexico and the United States) were chosen for estimating the reproductive ratio during the first four pandemic years. For each location, local epidemic seasons were defined based on the GMRF skyride plots generated above. To estimate the exponential growth rates (*r*_0_) of H1N1/2009 viruses, we analysed the virus sequence data that were collected only during the exponential phase of every seasonal epidemic (that is, before the peak date), see details of each location's exponential time spans in Supplementary Table 5. For each epidemic data set, an uncorrelated relaxed clock model with exponential coalescent and the SRD06 codon position model were used in BEAST. Four independent analyses were performed, each run for 10 million generations with sampling every 1,000 generations. We then used *r*_0_ to calculate the basic reproductive ratio (*R*_0_), which quantifies the transmission potential of viruses by averaging the number of secondary infections from one primary case in a host^1^,^19^.

### Discrete phylogeography analyses

To understand the spatial dynamics of H1N1/2009 viruses, phylogeographic analyses were performed in BEAST to reconstruct the ancestral geographical regions, diffusion rates and migration patterns simultaneously through time. Five different data sets were used to represent these global epidemic periods: 2009–2010, 2010–2011, 2011–2012, 2012–2013 and 2013–2014; and specifically from October to the following September of each year as inferred from Fig. 4a. The H1-HA sequences from each location with sufficient sequences for the analysis were downloaded from public resources, and the numbers of sequences were standardized for each location and time period to minimize the effect of geographical sampling bias. Nine geographical locations (that is, Africa, Australia, East Asia, Europe, the Middle East, North America, South America, South Asia and Southeast Asia) were selected and coded as discrete states. To estimate the diffusion rates among locations, the asymmetric substitution model with the Bayesian Stochastic Search Variable Selection option^66^ was used in BEAST to infer asymmetric diffusion rates between any pairwise location state, and also allowing BF calculations to test for significant diffusion rates. We used a strict clock model and exponential growth coalescent prior. Four independent analyses were performed using 100 million generations, with sampling every 10,000 generations. The resulting log files were used to calculate the BF for the diffusion between discrete locations, and to extract the actual non-zero rates and mean indicators for all statistically supported routes. Significant migration pathways were summarized based on the combination of both BF>3 and the mean indicator of >0.5. We defined the degree of rate support as follows: BF≥1,000 indicates decisive, 100≤BF<1,000 indicates very strong support, 10≤BF<100 indicates strong support and 3≤BF<10 indicates supported.

We also estimated the number of location state transitions (that is, Markov jump counts)^48^ using the asymmetric migration model described above, and plotted the total number of state counts for the migration in and out of each location. In our reconstruction of ancestral states we assume migration occurs at the tree nodes. We used the time between state changes (Markov reward) to estimate the duration the ancestral population occupies a particular location. We estimated the Markov rewards using the continuous-time Markov chain model^48^,^67^. Even though many individuals are infected each epidemic season, very few lineages persist to cause new epidemics. This persistent population forms the phylogenetic tree trunk^68^,^69^,^70^. To estimate the location and Markov rewards of the ancestral population that forms the phylogenetic tree trunk, we randomly sampled HA sequences from viruses isolated between 2009 and 2014 from the nine locations defined above, keeping at least 50 isolates per location per year where available. After removing identical sequences, our final data set contained 2,225 sequences. Four independent chains of 100 million generations sampled every 10,000 generations were run, following which the first 9,000 samples were excluded and the tree simulations combined. The trunk proportions were determined from the remaining 4,004 trees. If the tree trunk occupies a single location, then H1N1/2009 viruses would exhibit source–sink population dynamics^68^. Conversely, if multiple locations occupy the trunk, it would suggest that seasonal influenza emerges from a globally migrating population^69^,^70^.

### Genomic divergence and reassortment

Phylogenies of all eight gene segments using the global 485 data sets were generated in BEAST as described above for the analysis of H1N1/2009 temporal dynamics, with the exception that six independent analyses were performed per data set. To access the level of genomic reassortment, we used a multidimensional scaling of TMRCAs of each viral segment that were calculated from sets of the last 500 trees inferred in BEAST, and generated multidimensional scaling plots using the R statistics package. The TMRCAs of viruses circulating in each calendar year were also compared for all genes (Supplementary Table 9). Branches of the dated phylogenies for each gene are coloured according to different years of isolation (Supplementary Figs 8 and 9) or geographical locations (Supplementary Figs 9 and 10).

## Additional information

**Accession codes:** The sequence data have been deposited in GenBank database under accession codes KT180335 to KT182046.

**How to cite this article:** Su, Y. C. F. *et al*. Phylodynamics of H1N1/2009 influenza reveals the transition from host adaptation to immune-driven selection. *Nat. Commun.* 6:7952 doi: 10.1038/ncomms8952 (2015).

## Supplementary Material

Supplementary Information Supplementary Figures 1-10 and Supplementary Tables 1-9

## Figures and Tables

**Table 1:** Comparison of selection pressures of pandemic and post-pandemic periods for all genes of globally sampled H1N1/2009 viruses.

**Gene**	**Lineages**	**Branch d**_**N**_**/d**_**S**_ **by CODEML**			**Global d**_**N**_**/d**_**S**_ **by SLAC**	**Number of positively selected sites by M8+BEB (amino-acid position)**	**Number of positively selected sites by MEME (amino-acid position)**	**Number of sites under relaxed selection**
		**Internal**	**External**	**Internal/External**	**Mean (95% CI)**			
*PB2*	2009–2010	8.138	3.872	2.102	0.189 (0.171–0.209)	22 (23, 60*, 107, 127, 154*, 191*, 194*, 227, 251*, 255*, 288, 340*, 344*, 354, 456, 461*, 471, 480, 584, 588*, 648, 660*)	5 (4, 199, 227, 344, 373)	20
	2011–2014	0.109	0.1	1.089	0.121 (0.107–0.136)	11 (194, 195*, 221, 255*, 293*, 299*, 354, 456*, 464*, 588, 731*)	0 (0)	11
*PB1*	2009–2010	2.007	2.775	0.723	0.160 (0.144–0.178)	9 (257*, 353*, 435*, 563, 584, 587*, 609, 652*, 736*)	6 (207, 281, 387, 412, 608, 736)	8
	2011–2014	0.109	0.083	1.32	0.101 (0.089–0.114)	8 (20, 113*, 154*, 351, 397*, 435*, 645*, 652*)	4 (219, 231, 257, 633)	8
*PA*	2009–2010	4.569	2.646	1.727	0.202 (0.182–0.222)	8 (14*, 30, 321, 325, 343, 479*, 581*, 716*)	6 (14, 352, 387, 709, 711, 712)	7
	2011–2014	0.091	0.112	0.813	0.138 (0.123–0.155)	5 (14*, 100*, 266*, 330*, 343*)	2 (450, 574)	5
*HA*	2009–2010	0.41	0.288	1.424	0.345 (0.317–0.375)	20 (2, 49*, 114*, 142*, 145*, 154, 190, 200*, 202, 203*, 214, 220*, 239*, 266, 289*, 310*, 338*, 391*, 468*, 537)	12 (24, 49, 105, 145, 173, 391, 410, 436, 559, 560, 561, 563)	17
	2011–2014	0.224	0.223	1.005	0.328 (0.306–0.352)	16 (8*, 86*, 114*, 155*, 160*, 180*, 202*, 214*, 266*, 273*, 300*, 338*, 468*, 491*, 516*, 537*)	25 (26, 52, 65, 101, 114, 137, 142, 156, 158, 160, 173, 180, 188, 202, 214, 222, 224, 233, 239, 252, 273, 278, 303, 328, 465)	10
*NP*	2009–2010	11.423	4.176	2.736	0.113 (0.0976–0.130)	8 (22, 100*, 175, 197, 353*, 373*, 425*, 452)	2 (197, 301)	7
	2011–2014	0.058	0.065	0.894	0.103 (0.0865–0.121)	7 (22*, 34*, 71*, 373*, 400*, 425*, 498*)	5 (34, 208. 209, 301, 498)	5
*NA*	2009–2010	2.421	2.061	1.174	0.305 (0.275–0.336)	14 (43, 44*, 53, 80, 86, 106*, 241*, 248*, 275*, 308*, 313*, 332, 369*, 386*)	2 (35, 463)	14
	2011–2014	0.244	0.285	0.858	0.322 (0.291–0.354)	17 (34, 40, 41*, 43*, 44*, 45*, 82*, 200, 275*, 299, 313*, 321*, 339*, 386*, 389*, 396*, 397)	3 (151, 275, 386)	15
*M1*	2009–2010	0.074	0.146	0.51	0.138 (0.117–0.170)	2 (30, 80*)	1 (80)	1
	2011–2014	0.15	0.172	0.875	0.142 (0.112–0.177)	4 (80*, 191*, 242*, 250*)	0 (0)	4
*M2*	2009–2010	1.42	0.553	2.568	0.721 (0.568–0.900)	5 (10, 13, 27*, 82, 96)	0 (0)	5
	2011–2014	0.489	0.476	1.027	0.520 (0.392–0.673)	2 (13*, 21*)	0 (0)	2
*NS1*	2009–2010	0.269	0.469	0.573	0.539 (0.475–0.608)	4 (55*, 90*, 93*, 123*)	2 (116,178)	4
	2011–2014	0.291	0.241	1.208	0.416 (0.358–0.481)	8 (55*, 90*, 115, 122*, 131, 186*, 191*, 205*)	0 (0)	8
*NS2*	2009–2010	Inf	0.319	—	0.350 (0.278–0.434)	4 (20*, 89*, 115, 120*)	1(120)	3
	2011–2014	0.325	0.305	1.064	0.333 (0.252–0.429)	6 (29, 34, 48, 60, 89, 115)	0 (0)	6

Sites with asterisks (*) indicate statistically significant with posterior probability of ≥0.95. Inf indicates zero synonymous changes and this value equals infinity.

**Table 2:** TMRCA estimates and nucleotide substitution rates for all gene segments of globally sampled H1N1/2009 viruses from 2009–2014.

**Gene**	**TMRCA of root height (mean)**	**Upper 95% HPD**	**Lower 95% HPD**	**Mean substitution rate**	**Lower 95% HPD**	**Upper 95% HPD**
*PB2*	2009.040 (16 January 2009)	2008.941 (11 December 2008)	2009.139 (21 February 2009)	3.94E−03	3.50E−03	4.42E−03
*PB1*	2008.999 (31 December 2008)	2008.890 (22 November 2008)	2009.103 (8 February 2009)	3.74E−03	3.31E−03	4.45E−03
*PA*	2008.981 (25 December 2008)	2008.875 (17 November 2008)	2009.091 (4 February 2009)	3.77E−03	3.33E−03	4.20E−03
*HA*	2009.031 (13 January 2009)	2008.931 (7 December 2008)	2009.131 (18 February 2009)	5.34E−03	4.76E−03	5.95E−03
*NP*	2009.041 (16 January 2009)	2008.915 (1 December 2008)	2009.150 (25 February 2009)	3.92E−03	3.34E−03	4.46E−03
*NA*	2009.081 (31 January 2009)	2008.982 (26 December 2008)	2009.177 (7 March 2009)	5.21E−03	4.64E−03	5.82E−03
*MP*	2009.071 (27 January 2009)	2008.941 (11 December 2008)	2009.191 (12 March 2009)	3.52E−03	2.98E−03	4.09E−03
*NS*	2009.051 (20 January 2009)	2008.921 (4 December 2008)	2009.171 (5 March 2009)	4.50E−03	3.80E−03	5.20E−03
