# Supplementary material for: Phylodynamics of H1N1/2009 influenza reveals the transition from host adaptation to immune-driven selection
Source: Nat Commun. 2015 Aug 6;6:7952. doi: 10.1038/ncomms8952 (PMC4918339; doi:10.1038/ncomms8952)
Supplement: Supplementary Information — Supplementary Figures 1-10 and Supplementary Tables 1-9 [file ncomms8952-s1.pdf]

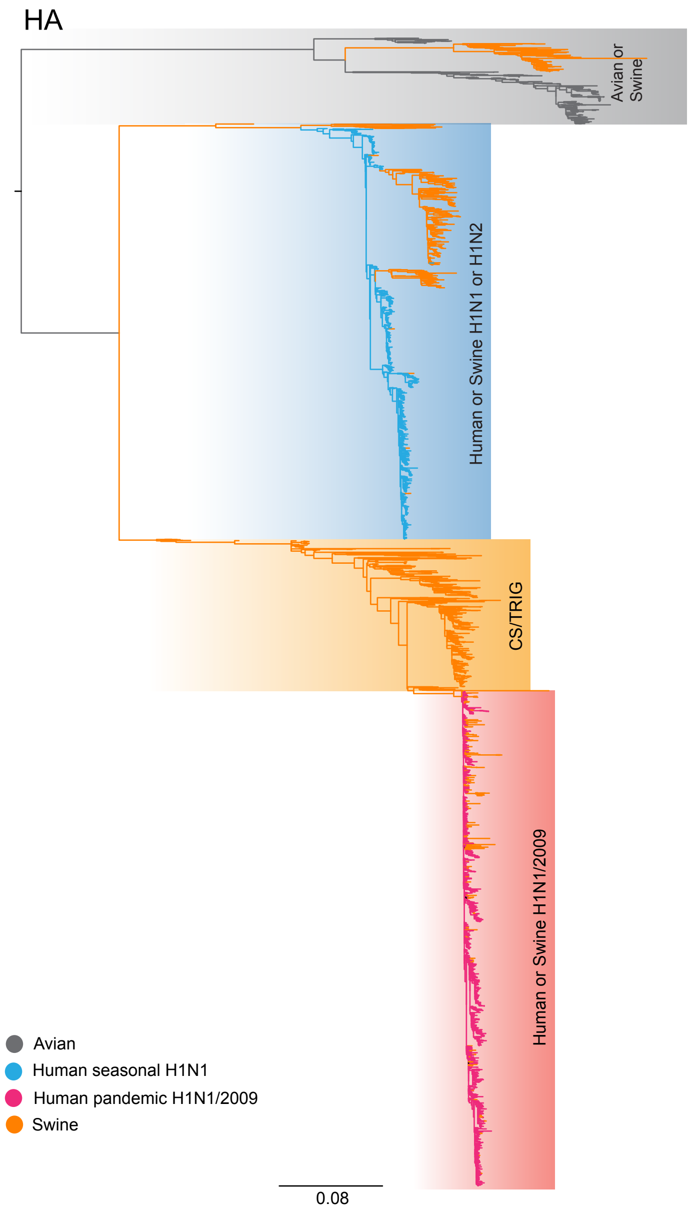

Supplementary Fig. 1. Phylogeny of influenza A (H1) viruses from 1930 to 2014. Global maximum likelihood phylogeny of 5804 H1-HA sequences of representative avian (dark grey branches), human (blue branches represent human seasonal H1N1 subtype viruses; red branches denote human pandemic H1N1/2009 viruses), and swine (orange branches) viruses. CS is classical swine and TRIG is triple-reassortant swine. Scale bar represents number of substitutions per site.

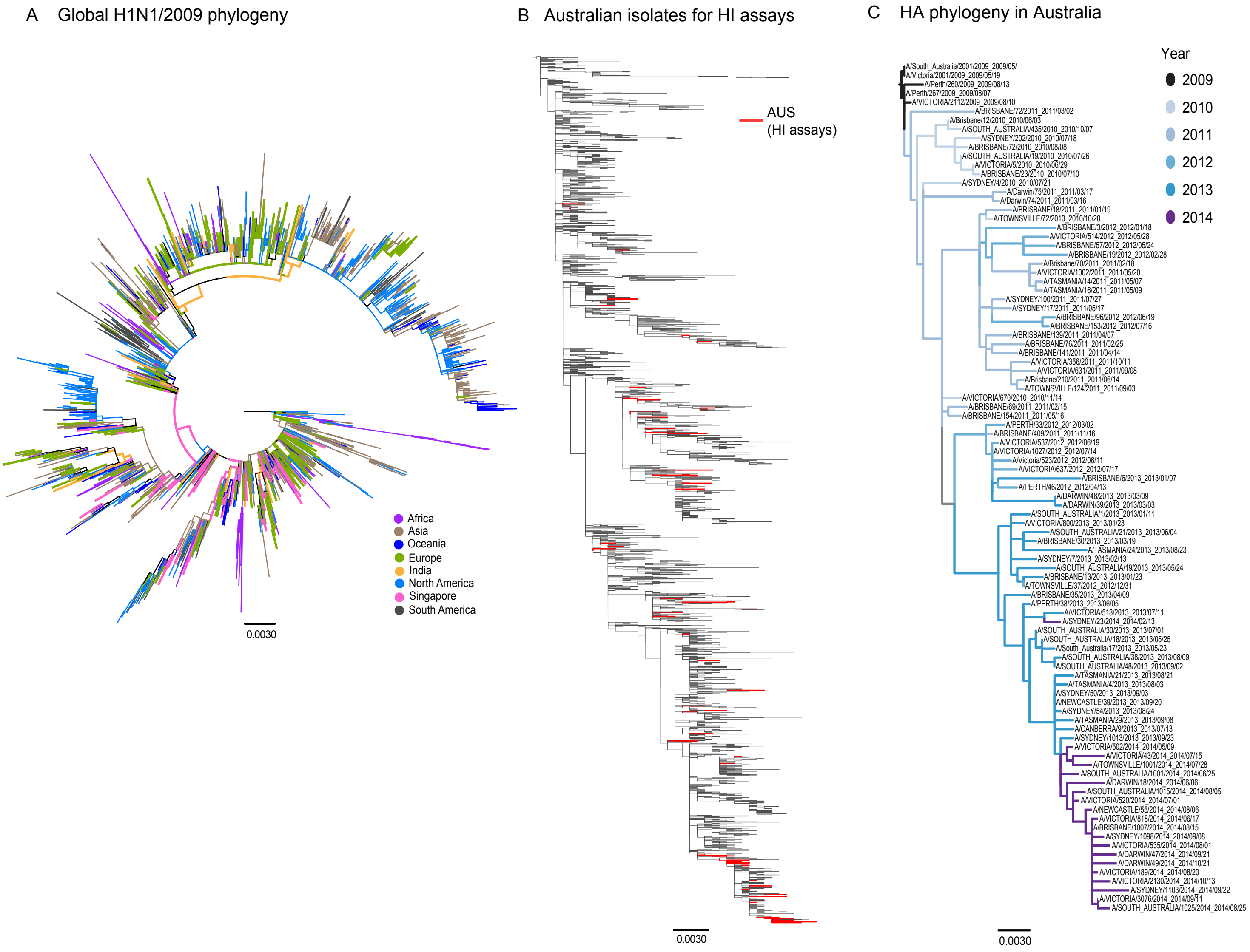

Supplementary Fig. 2. Maximum-likelihood phylogenies of human H1N1/2009 viruses. A. Global phylogeny of 2280 human H1 HA sequences during 2009–2014, coloured branches represent different geographical regions. B. Representative Australian strains (2009–2014) used in HI assays are marked by red branches on the global phylogenetic tree. C. Phylogeny of selected H1-HA for viruses isolated in Australia from 2009–2014 with branches coloured by year of virus isolation. Scale bars represent number of substitutions per site.

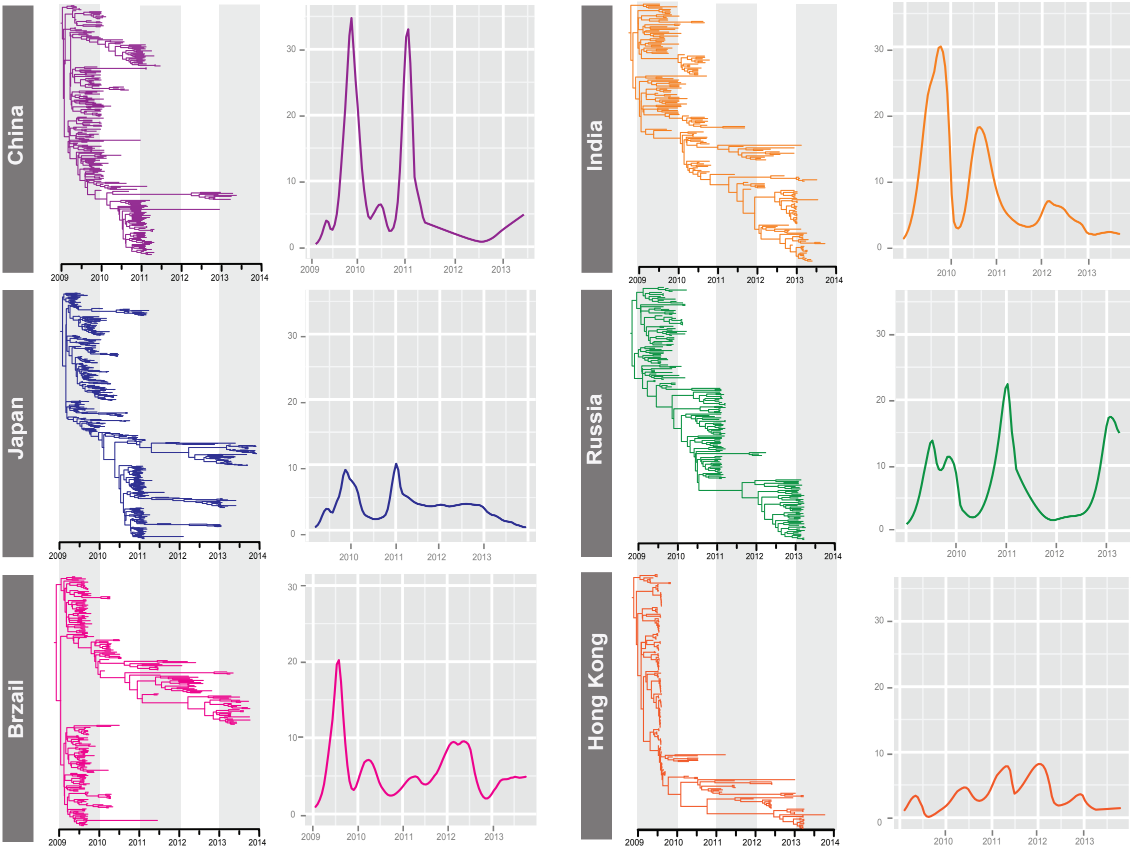

Supplementary Fig. 3. Comparative phylogenetic analyses and population dynamics of H1-HA viruses circulating in different geographical regions. Phylogenies were inferred using the uncorrelated lognormal relaxed clock model and relative genetic diversity estimated using a Gaussian Markov Random Field (GMRF) coalescent prior. Solid lines in the GMRF plot represent the mean relative genetic diversity over time.

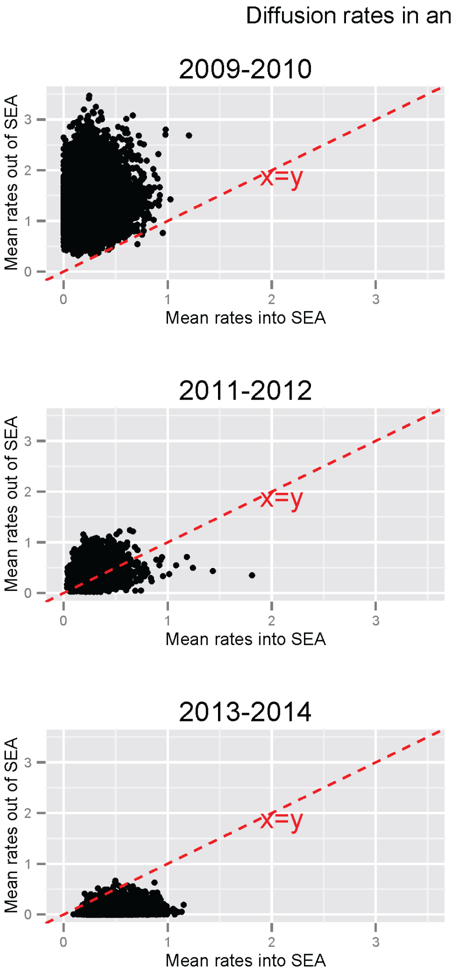

Supplementary Fig. 4. Scatter plots of the mean migration rate showing the relationship of the in- and out-migration of Southeast Asia in each epidemic year. For every MCMC state, the mean actual rate of diffusion from Southeast Asia to all other localities (y-axis) versus the mean actual rate from all other localities into Southeast Asia (x-axis) is plotted for all MCMC states after burn-in. The red diagonal line indicates the mean rates of migration in and out of Southeast Asia are identical.

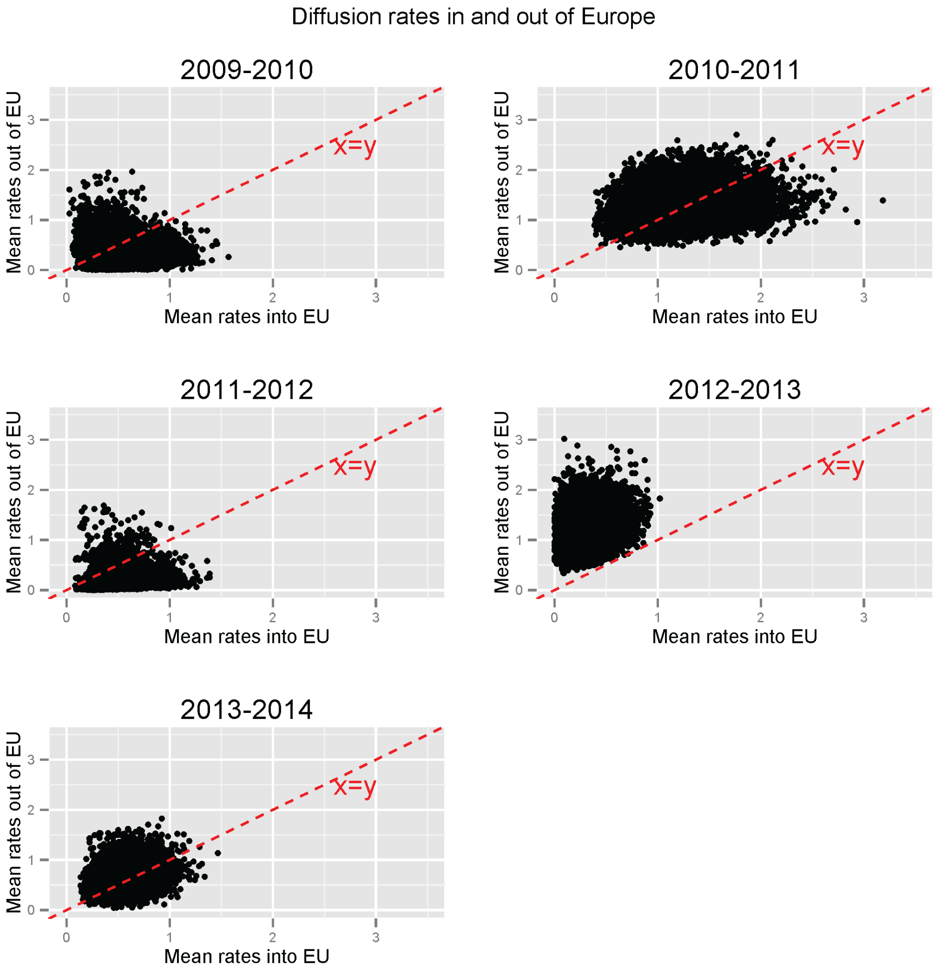

Supplementary Fig. 5. Scatter plots of the mean migration rate showing the relationship of the in- and out-migration of Europe in each epidemic year. For every MCMC state, the mean actual rate of diffusion from Europe to all other localities (y-axis) versus the mean actual rate from all other localities into Europe (x-axis) is plotted for all MCMC states after burn-in. The red diagonal line indicates the mean rates of migration in and out of Europe are identical.

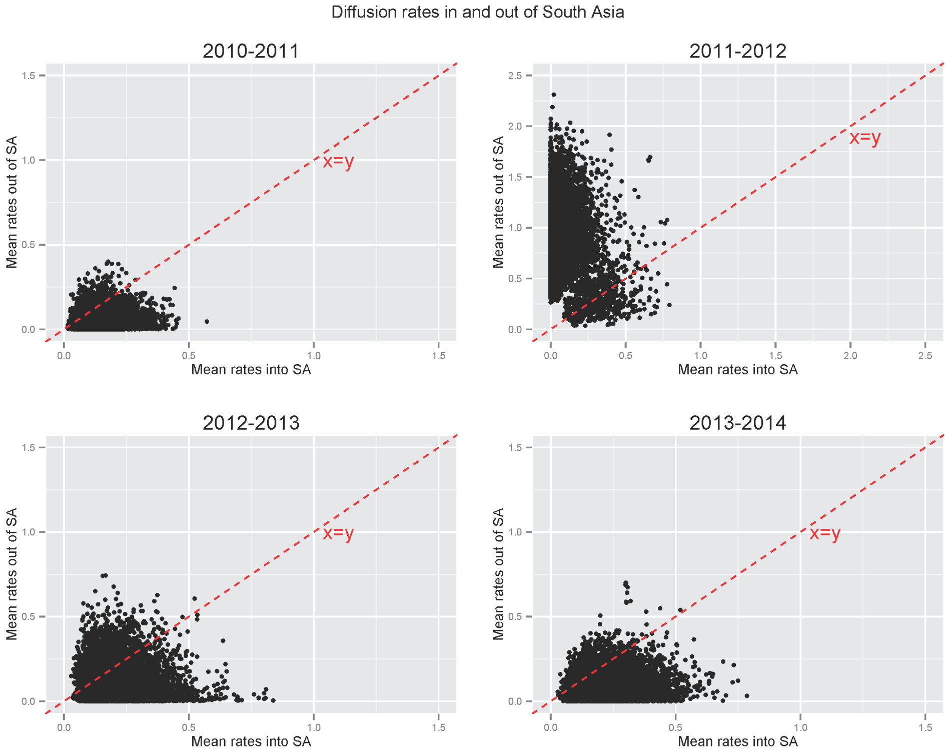

Supplementary Fig. 6. Scatter plots of the mean migration rate showing the relationship of the in- and out-migration of South Asia in each epidemic year. For every MCMC state, the mean actual rate of diffusion from South Asia to all other localities (y-axis) versus the mean actual rate from all other localities into South Asia (x-axis) is plotted for all MCMC states after burn-in. The red diagonal line indicates the mean rates of migration in and out of South Asia are identical.

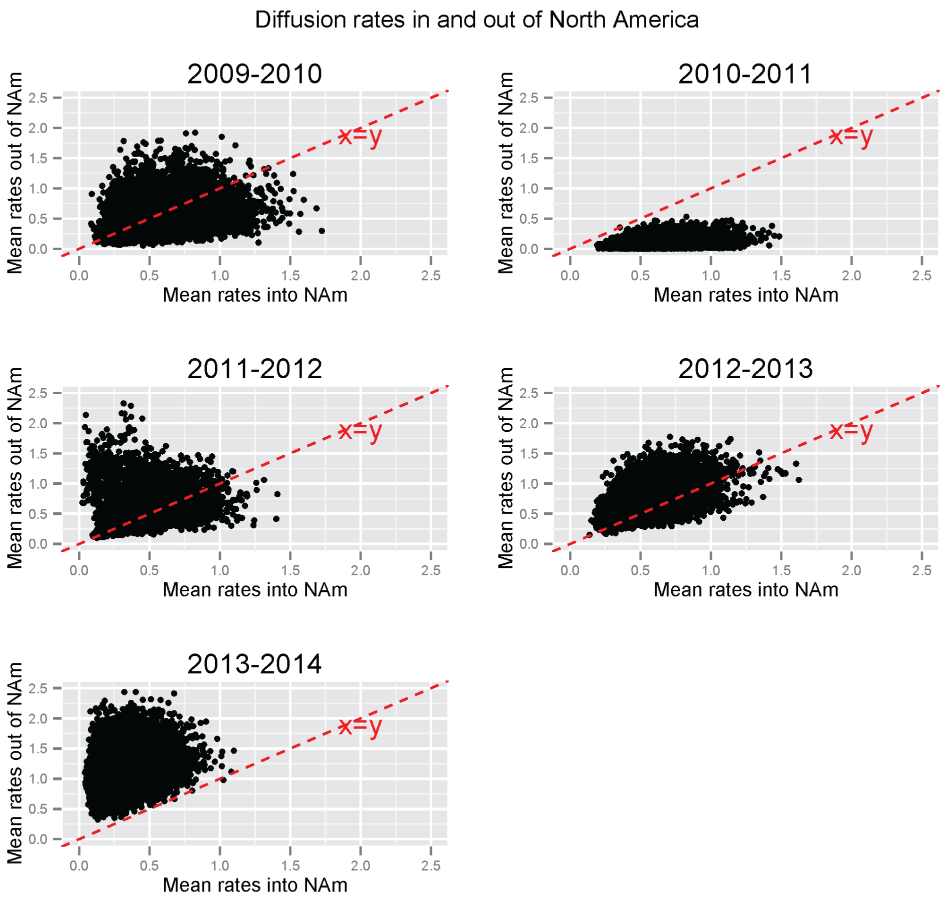

Supplementary Fig. 7. Scatter plots of the mean migration rate showing the relationship of the in- and out-migration of North America in each epidemic year. For every MCMC state, the mean actual rate of diffusion from North America to all other localities (y-axis) versus the mean actual rate from all other localities into North America (x-axis) is plotted for all MCMC states after burn-in. The red diagonal line indicates the mean rates of migration in and out of North America are identical.

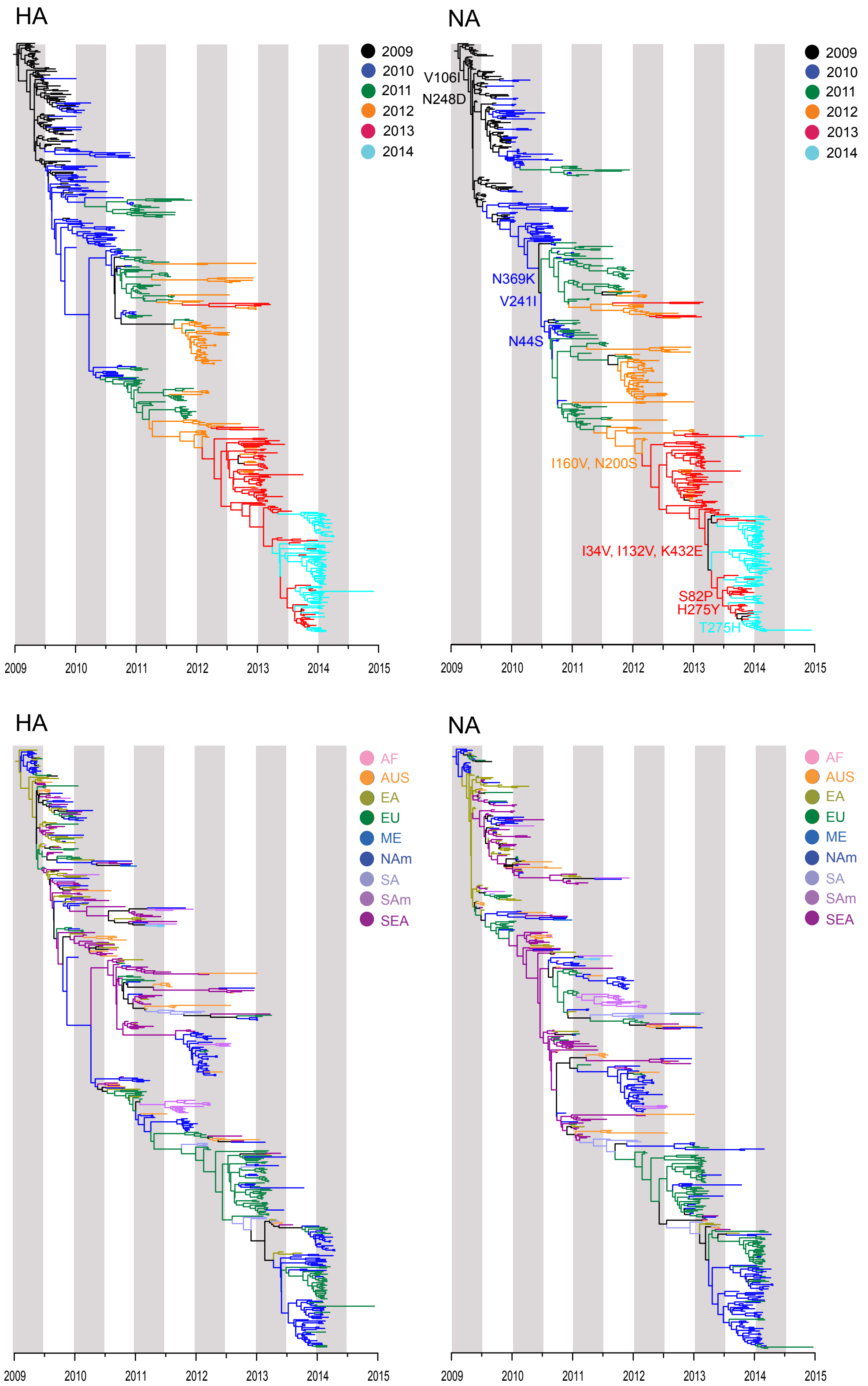

Supplementary Fig. 8. Dated HA and NA phylogenies of human H1N1/2009 viruses during 2009–2014. Phylogenies were inferred using the uncorrelated lognormal relaxed clock model and a Gaussian Markov Random Field (GMRF) coalescent prior. Representative amino acid substitutions are mapped at the major tree nodes (see Fig. 1 for the HA gene). Coloured branches represent different year of isolation (upper panels) and geographical locations (lower panels). Representative amino acid substitutions are mapped at the major tree nodes.

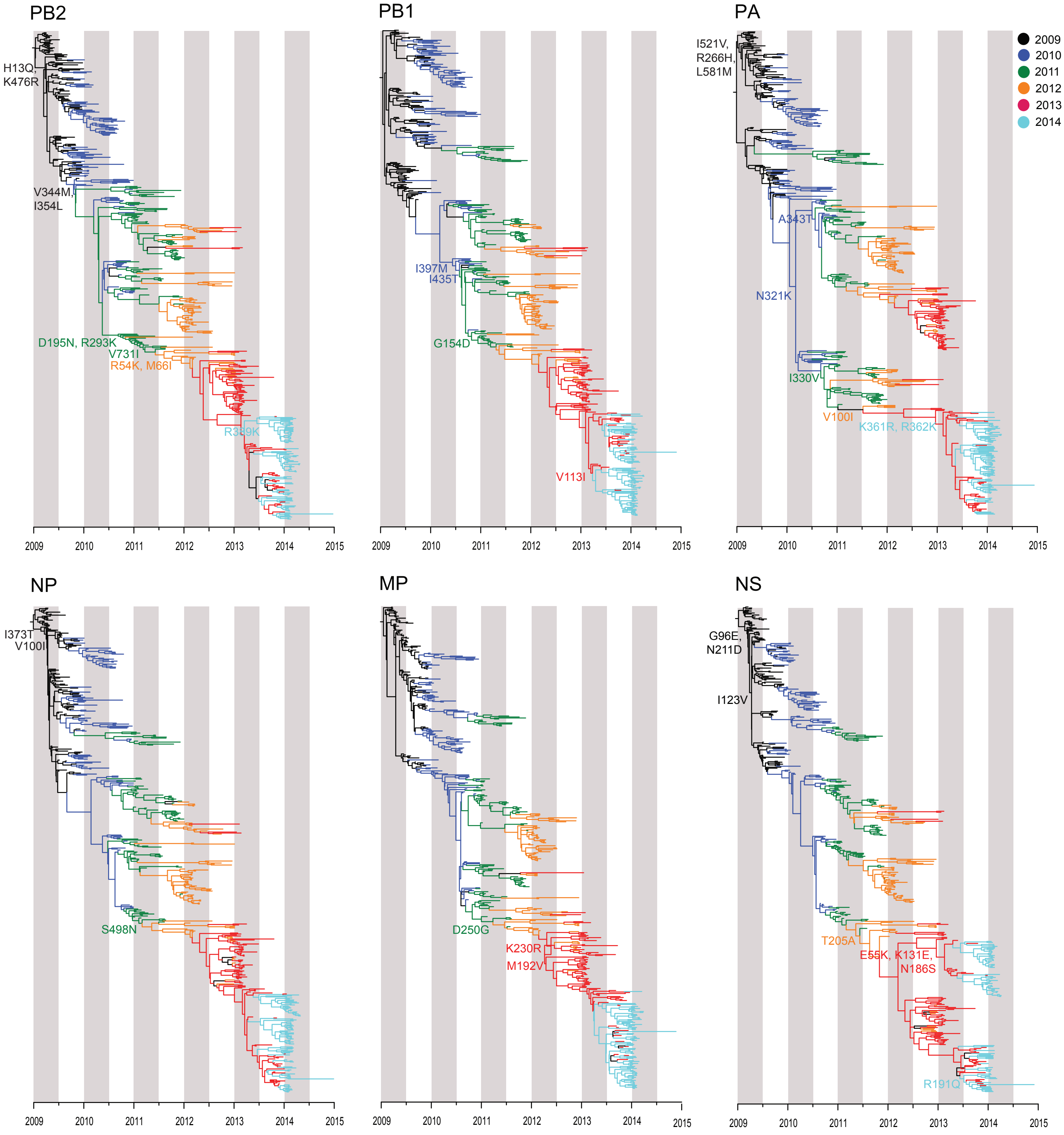

Supplementary Fig. 9. Divergence times and evolutionary relationships of the internal genes of human H1N1/2009 viruses during 2009–2014. Dated phylogenies of six internal genes are in this order: PB2, PB1, PA, NP, MP and NS. Phylogenies were inferred using the uncorrelated lognormal relaxed clock model and a Gaussian Markov Random Field (GMRF) coalescent prior. Representative amino acid substitutions are mapped at the major tree nodes. Coloured branches represent different year of isolation.

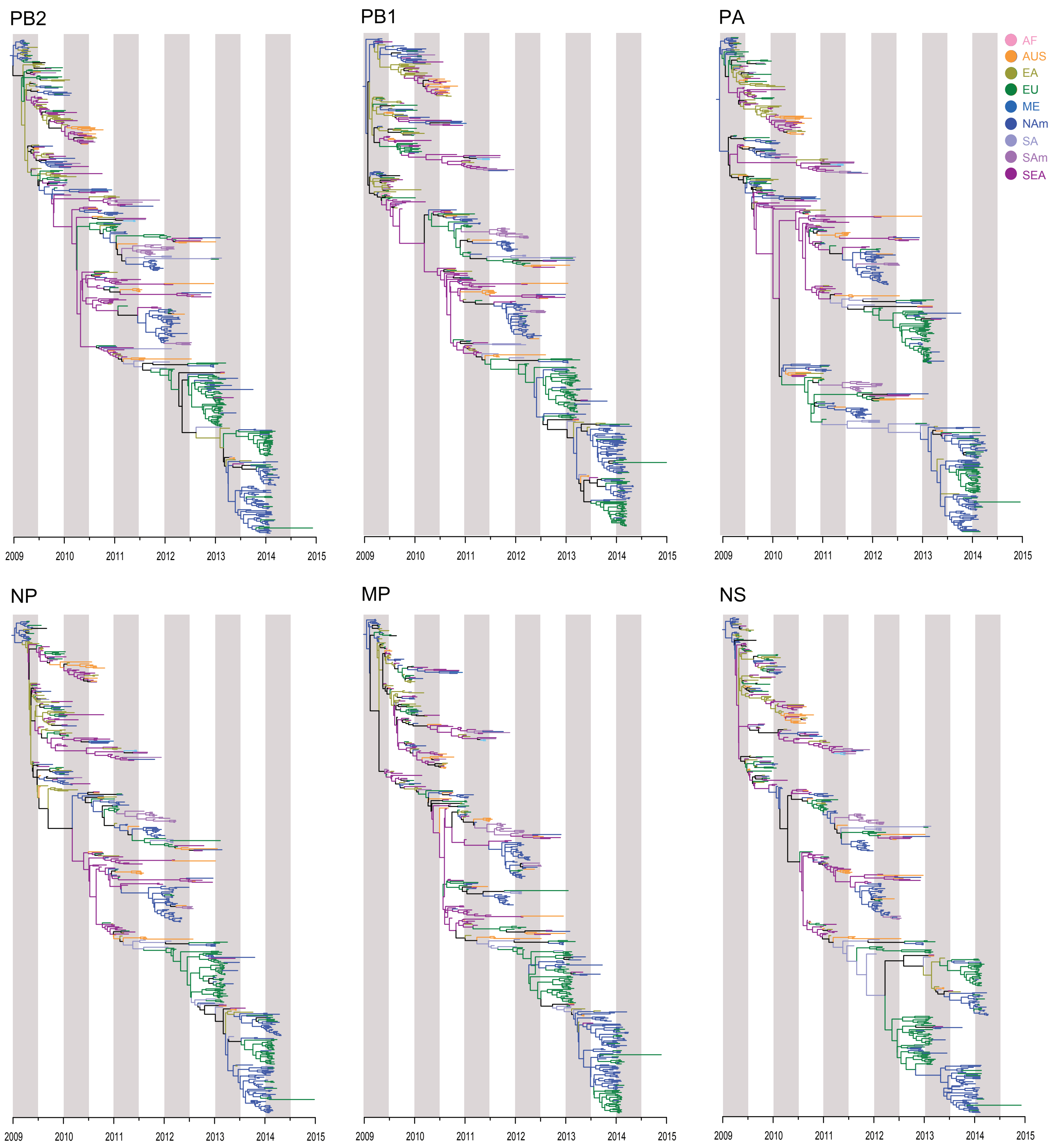

Supplementary Fig. 10. Divergence times and evolutionary relationships of the internal genes of human H1N1/2009 viruses during 2009–2014. Dated phylogenies of six internal genes are identical to Supplementary Fig 9, except coloured branches denote different geographical locations.

Supplementary Tables

Supplementary Table 1. Selection pressure acting on globally sampled H1N1/2009 genes (2009–2014). For each individual gene, the branch and global d_N/d_S were calculated using CODEML, SLAC and MEME methods.

Gene	Branch d_N/d_S (CODEML)			Global d_N/d_S (SLAC)			Number of positively selected sites by MEME (amino acid position)
	Internal	External	Internal/external	Mean	Lower (95% CI)	Upper (95% CI)	
**PB2**	0.067	0.098	0.689	0.117	0.104	0.131	0 (0)
**PB1**	0.129	0.096	1.342	0.114	0.101	0.128	1 (485)
**PA**	0.091	0.114	0.803	0.136	0.121	0.152	2 (261,574)
**HA**	0.222	0.214	1.035	0.262	0.238	0.287	2 (180,239)
**NP**	0.062	0.064	0.968	0.087	0.072	0.103	0 (0)
**NA**	0.299	0.268	1.114	0.328	0.296	0.361	3 (275,313,463)
**M1**	0.269	0.156	1.728	0.148	0.118	0.182	0 (0)
**M2**	1.099	0.557	1.971	0.519	0.393	0.668	0 (0)
**NS1**	0.229	0.243	0.943	0.451	0.390	0.519	0 (0)
**NS2**	0.465	0.287	1.622	0.282	0.210	0.369	0 (0)

Supplementary Table 2. Yearly d_N/d_S ratio estimates of the H1N1/2009 virus. The d_N/d_S ratios were estimated for each gene in different years by SLAC method.

Gene	Year	Global d_N/d_S		
		Mean	Lower (95% CI)	Upper (95% CI)
**PB2**	2009	0.207	0.18	0.236
	2010	0.105	0.0957	0.116
	2011	0.0868	0.0755	0.0993
	2012	0.061	0.0512	0.072
	2013	0.0965	0.0813	0.114
	2014	0.102	0.0834	0.124
**PB1**	2009	0.151	0.128	0.176
	2010	0.0929	0.0842	0.102
	2011	0.0916	0.0802	0.104
	2012	0.065	0.0541	0.0772
	2013	0.0885	0.0732	0.106
	2014	0.0637	0.0512	0.0781
**PA**	2009	0.2	0.171	0.232
	2010	0.109	0.0982	0.121
	2011	0.115	0.101	0.131
	2012	0.0783	0.0656	0.0925
	2013	0.134	0.112	0.159
	2014	0.14	0.116	0.168
**HA**	2009	0.370	0.337	0.406
	2010	0.312	0.285	0.341
	2011	0.306	0.282	0.332
	2012	0.303	0.277	0.330
	2013	0.275	0.250	0.301
	2014	0.207	0.183	0.232
**NA**	2009	0.28	0.234	0.331

	2010	0.326	0.295	0.359
	2011	0.406	0.371	0.444
	2012	0.437	0.397	0.48
	2013	0.369	0.336	0.403
	2014	0.354	0.317	0.393
**M1**	2009	0.136	0.0934	0.189
	2010	0.148	0.122	0.177
	2011	0.0761	0.0536	0.104
	2012	0.083	0.0629	0.107
	2013	0.0726	0.0481	0.104
	2014	0.0487	0.0328	0.069
**M2**	2009	0.570	0.389	0.799
	2010	0.743	0.600	0.908
	2011	0.382	0.255	0.545
	2012	0.492	0.380	0.625
	2013	0.438	0.280	0.648
	2014	0.554	0.415	0.720
**NS1**	2009	0.466	0.377	0.569
	2010	0.314	0.276	0.357
	2011	0.34	0.293	0.392
	2012	0.269	0.223	0.322
	2013	0.319	0.262	0.394
	2014	0.354	0.281	0.437
**NS2**	2009	0.381	0.270	0.520
	2010	0.313	0.254	0.382
	2011	0.223	0.169	0.289
	2012	0.178	0.102	0.284
	2013	0.316	0.210	0.436
	2014	0.468	0.308	0.677

Supplementary Table 3. Antigenic characterization of H1N1/2009 viruses using haemagglutinin inhibition (HI) assays. Antisera raised in ferrets were used to test against a range of H1H1/2009 viruses from Australia collected from 2009–2014.

**Virus strains**	**Antisera**					
	**CAL/7**	**ILLINOIS/9**	**CHCH/16**	**BRIS/70**	**VIC/637**	**STH_AUS/17**
A/CALIFORINA/07/2009 (reference)	5120	5120	2560	2560	5120	640
A/PERTH/260/2009	2560	2560	1280	2560	2560	640
A/SOUTH AUSTRALIA/2001/2009	2560	2560	1280	2560	2560	640
A/VICTORIA/2112/2009	2560	2560	1280	2560	2560	640
A/BRISBANE/12/2010	5120	2560	10240	5120	5120	1280
A/BRISBANE/23/2010	5120	2560	10240	5120	5120	1280
A/SOUTH AUSTRALIA/19/2010	5120	2560	10240	5120	5120	1280
A/SYDNEY/202/2010	2560	1280	5120	1280	2560	640
A/SYDNEY/4/2010	5120	2560	10240	10240	5120	1280
A/TOWNSVILLE/72/2010	320	80	320	160	640	160
A/VICTORIA/670/2010	2560	2560	1280	2560	2560	640
A/BRISBANE/139/2011	320	320	640	160	640	160
A/BRISBANE/154/2011	320	80	320	160	320	160
A/BRISBANE/18/2011	160	80	320	160	640	160
A/BRISBANE/210/2011	320	160	320	160	640	160
A/BRISBANE/409/2011	2560	640	1280	1280	1280	320
A/BRISBANE/72/2011	2560	2560	2560	5120	2560	640
A/BRISBANE/76/2011	160	80	160	160	320	80
A/DARWIN/74/2011	5120	5120	5120	5120	5120	1280
A/SYDNEY/100/2011	2560	1280	1280	2560	2560	640
A/TASMANIA/14/2011	320	160	320	320	640	160
A/VICTORIA/1002/2011	1280	1280	640	1280	1280	320
A/VICTORIA/356/2011	2560	1280	1280	1280	1280	320
A/BRISBANE/3/2012	640	320	640	640	640	320

A/BRISBANE/57/2012	160	80	160	160	320	80
A/BRISBANE/96/2012	320	160	640	320	640	320
A/PERTH/33/2012	1280	1280	640	1280	1280	320
A/PERTH/46/2012	320	80	320	320	640	80
A/TOWNSVILLE/37/2012	2560	1280	5120	2560	2560	640
A/VICTORIA/514/2012	2560	2560	1280	2560	2560	640
A/VICTORIA/523/2012	1280	640	640	1280	1280	320
A/VICTORIA/537/2012	2560	2560	2560	2560	2560	640
A/VICTORIA/637/2012	1280	1280	640	1280	2560	320
A/BRISBANE/13/2013	320	80	320	160	640	160
A/BRISBANE/30/2013	160	0	160	80	320	80
A/BRISBANE/35/2013	5120	5120	5120	5120	5120	1280
A/BRISBANE/6/2013	160	80	320	160	320	80
A/CANBERRA/9/2013	5120	5120	5120	5120	5120	1280
A/DARWIN/39/2013	320	80	320	320	1280	80
A/PERTH/38/2013	5120	5120	5120	5120	5120	1280
A/SOUTH AUSTRALIA/17/2013	10240	5120	5120	10240	5120	1280
A/SOUTH AUSTRALIA/19/2013	1280	1280	640	1280	1280	320
A/SOUTH AUSTRALIA/38/2013	2560	2560	2560	2560	2560	640
A/SOUTH AUSTRALIA/48/2013	2560	2560	2560	2560	2560	640
A/SYDNEY/1013/2013	5120	5120	5120	10240	5120	1280
A/SYDNEY/54/2013	5120	5120	5120	5120	5120	1280
A/TASMANIA/24/2013	5120	5120	5120	5120	5120	1280
A/TASMANIA/4/2013	10240	10240	10240	10240	5120	2560
A/VICTORIA/518/2013	5120	5120	5120	5120	5120	1280
A/VICTORIA/800/2013	2560	2560	1280	2560	2560	640
A/BRISBANE/1007/2014	2560	2560	2560	2560	2560	640
A/DARWIN/18/2014	2560	2560	1280	2560	2560	640
A/DARWIN/47/2014	10240	5120	5120	5120	5120	1280
A/DARWIN/49/2014	2560	2560	1280	2560	2560	640

A/NEWCASTLE/55/2014	1280	1280	1280	1280	1280	320
A/SOUTH AUSTRALIA/1015/2014	2560	2560	2560	2560	2560	640
A/SOUTH AUSTRALIA/1025/2014	5120	2560	2560	2560	5120	1280
A/SYDNEY/1098/2014	5120	5120	2560	5120	5120	1280
A/SYDNEY/1103/2014	5120	5120	5120	5120	5120	1280
A/TOWNSVILLE/1001/2014	2560	2560	2560	2560	2560	640
A/VICTORIA/189/2014	5120	5120	2560	5120	5120	1280
A/VICTORIA/2130/2014	2560	2560	2560	2560	2560	640
A/VICTORIA/3076/2014	2560	2560	2560	2560	2560	640
A/VICTORIA/43/2014	2560	2560	1280	2560	2560	640
A/VICTORIA/502/2014	1280	1280	1280	1280	1280	640
A/VICTORIA/520/2014	5120	5120	5120	5120	5120	1280
A/VICTORIA/535/2014	5120	5120	5120	5120	5120	1280

Supplementary Table 4. Regional GMRF skyride analyses of H1N1/2009 virus, 2009–2014. The TMRCAs and occurrences of epidemic peaks of H1N1/2009 HA viruses were estimated for each selected location.

Location	TMRCA of root height	Upper 95% HPD	Lower 95% HPD	2009–2010	2010–2011	2011–2012	2012–2013	2013–2014
**Australia** (511 sequences)	2009.119	2009.006	2009.223	16 Jul 2009	30 Jul 2010	30 Jul 2011	-	10 Sep 2013
**Brazil** (239 sequences)	2008.849	2008.650	2009.052	19 Jul 2009	24 Mar 2010	4 Apr 2011	12 Mar 2012	-
**China** (249 sequences)	2009.044	2008.884	2009.185	9 Dec 2009	10 Jun 2010	11 Jan 2011	-	-
**Europe** (629 sequences)	2009.048	2008.940	2009.150	24 Nov 2009	-	01 Jan 2011	13 Jan 2012	1 Jan 2013
**Hong Kong** (225 sequences)	2008.886	2008.699	2009.055	10 May 2009	3 Jun 2010	10 May 2011	17 Feb 2012; 8 Dec 2012	-
**India** (194 sequences)	2008.748	2008.518	2008.976	9 Oct 2009	24 Aug 2010	-	28 Feb 2012	-
**Japan** (641 sequences)	2009.085	2008.972	2009.188	20 Jun 2009; 13 Nov 2009	-	1 Jan 2011	-	-
**Mexico** (239 sequences)	2008.810	2008.653	2008.954	26 Mar 2009; 22 Oct 2009	22 Oct 2010	18 Jun 2011	13 Aug 2012	18 Jun 2013
**Russia** (196 sequences)	2008.782	2008.529	2009.006	13 Jul 2009; 28 Oct 2009	-	11 Jan 2011	-	27 Jan 2013
**Singapore** (348 sequences)	2009.101	2008.979	2009.221	20 Aug 2009; 29 Nov 2009	13 May 2010; 24 Sep 2010	13 Feb 2011	13 May 2012	-
**South America** (489 sequences)	2009.066	2008.950	2009.179	16 Aug 2009	20 Feb 2010	26 Jun 2011	20 Feb 2012	1 Jun 2013
**United States** (630 sequences)	2009.018	2008.919	2009.112	18 Nov 2009	-	01 Jan 2011	28 Feb 2012	14 Feb 2013

Supplementary Table 5. Estimation of reproductive ratio (R_0). Approximate time phases of exponential growth and the estimated mean basic reproductive ratio (R_0) in regions representing the Tropics, the Northern and Southern Hemispheres. Values in bracket represent 95% highest posterior density (HPD) intervals.

Location	2009–2010 exponential phase	mean R_0 (95% confidence interval)	2010–2011 exponential phase	mean R_0 (95% confidence interval)	2011–2012 exponential phase	mean R_0 (95% confidence interval)	2012–2013 exponential phase	mean R_0 (95% confidence interval)	2013–2014 exponential phase	mean R_0 (95% confidence interval)
**Australia**	Apr–Jul 2009	1.00 (0.99–1.03)	Mar–Jul 2010	1.03 (1.01–1.05)	Apr–Jul 2011	1.03 (1.00–1.03)	-	-	Oct 2012–Mar 2013	1.03 (0.97–1.11)
**Europe**	Apr–Nov 2009	1.05 (1.04–1.07)	Aug 2010–Jan 2011	1.02 (1.01–1.03)	Nov 2011–Jan 2012	1.00 (0.98–1.03)	Jun 2012–Jan 2013	1.00 (0.99–1.01)	-	-
**Singapore**	May–Aug 2009	1.10 (1.05–1.16)	Feb–May 2010	1.03 (1.00–1.05)	Nov 2010–Feb 2011	1.01 (1.00–1.03)	Aug 2011–May 2012	1.01 (0.97–1.03)	-	-
**Mexico**	Mar 2009; Jul–Oct 2009	1.08 (0.87–1.33); 1.02 (0.98–1.07)	Feb 2010–Nov 2010	1.02 (0.98–1.05)	Sep 2011–Aug 2012	1.01 (1.00–1.04)	Jan–Jun 2013	1.11 (1.04–1.19)	Jan–Apr 2014	1.02 (1.00–1.05)
**USA**	Apr–Nov 2009	1.05 (1.04–1.07)	Sep 2010–Jan 2011	1.01 (0.99–1.02)	Dec 2011–Feb 2012	1.01 (0.99–1.02)	Aug 2012–Feb 2013	1.00 (0.99–1.01)	Aug 2013–Jan 2014	1.03 (1.02–1.04)

Supplementary Table 6. Trunk reward proportion (unit as years) for each geographic location during 2009–2014. Nine geographical locations were coded as discrete states and the Markov rewards were estimated through time using the continuous-time Markov chain (CTMC) model in BEAST.

Location	Slice Time	Total trunk reward proportion (mean)	Standard deviation
Africa	2009.5	0	0
East Asia	2009.5	0.05803	0.21876
Europe	2009.5	0.02344	0.12759
North America	2009.5	0.45864	0.48046
Australia	2009.5	0	0
Southeast Asia	2009.5	0.10029	0.29347
South America	2009.5	0.35793	0.45523
South Asia	2009.5	0.0005	0.0111
Middle East	2009.5	0	0
Africa	2009.75	0	0
East Asia	2009.75	0.18443	0.3326
Europe	2009.75	0.15834	0.32806
North America	2009.75	0.41514	0.45269
Australia	2009.75	0.01685	0.10697
Southeast Asia	2009.75	0.18238	0.35568
South America	2009.75	0.04039	0.17018
South Asia	2009.75	0.00726	0.06414
Middle East	2009.75	0.00305	0.03577
Africa	2010	0.00086	0.01187
East Asia	2010	0.27896	0.35299
Europe	2010	0.0715	0.20945
North America	2010	0.0726	0.2044
Australia	2010	0.02095	0.10411
Southeast Asia	2010	0.5066	0.38678

South America	2010	0.00732	0.05643
South Asia	2010	0.01177	0.08589
Middle East	2010	0.00756	0.05959
Africa	2010.25	0	0
East Asia	2010.25	0.05084	0.18448
Europe	2010.25	0.0194	0.11724
North America	2010.25	0.07144	0.2103
Australia	2010.25	0.03088	0.15306
Southeast Asia	2010.25	0.78528	0.35868
South America	2010.25	0.00296	0.03975
South Asia	2010.25	0.03705	0.1609
Middle East	2010.25	0.00143	0.03195
Africa	2010.5	0	0
East Asia	2010.5	0.01246	0.08546
Europe	2010.5	0.16382	0.32181
North America	2010.5	0.61744	0.45356
Australia	2010.5	0.07707	0.24456
Southeast Asia	2010.5	0.11108	0.26769
South America	2010.5	0.00179	0.02823
South Asia	2010.5	0.0085	0.0733
Middle East	2010.5	0.00435	0.04685
Africa	2010.75	0	0
East Asia	2010.75	0.00177	0.02806
Europe	2010.75	0.36071	0.44626
North America	2010.75	0.62431	0.4506
Australia	2010.75	0.00238	0.0308
Southeast Asia	2010.75	0.00109	0.02218
South America	2010.75	0	0
South Asia	2010.75	0.00014	0.00318
Middle East	2010.75	0.00187	0.04196

Africa	2011	0.00109	0.01785
East Asia	2011	0.00431	0.04977
Europe	2011	0.32452	0.42251
North America	2011	0.52522	0.47799
Australia	2011	0.00403	0.04557
Southeast Asia	2011	0.00059	0.00943
South America	2011	0	0
South Asia	2011	0.13671	0.27484
Middle East	2011	0.00027	0.00603
Africa	2011.25	0	0
East Asia	2011.25	0	0
Europe	2011.25	0.02903	0.13581
North America	2011.25	0.0102	0.08263
Australia	2011.25	0.00045	0.01009
Southeast Asia	2011.25	0.00035	0.00788
South America	2011.25	0.00138	0.0232
South Asia	2011.25	0.96237	0.15625
Middle East	2011.25	0.00066	0.01124
Africa	2011.5	0.00078	0.01649
East Asia	2011.5	0.00111	0.0159
Europe	2011.5	0.07614	0.20212
North America	2011.5	0.01282	0.07981
Australia	2011.5	0.00249	0.04154
Southeast Asia	2011.5	0.0079	0.06696
South America	2011.5	0.00058	0.01303
South Asia	2011.5	0.90355	0.23933
Middle East	2011.5	0.00102	0.01967
Africa	2011.75	0.00078	0.01649
East Asia	2011.75	0.00157	0.01733
Europe	2011.75	0.28617	0.31657

North America	2011.75	0.03115	0.12755
Australia	2011.75	0.00874	0.06967
Southeast Asia	2011.75	0.01018	0.07386
South America	2011.75	0.00497	0.05171
South Asia	2011.75	0.66048	0.33764
Middle East	2011.75	0.00538	0.04815
Africa	2012	0.00004	0.001
East Asia	2012	0.00406	0.04911
Europe	2012	0.50839	0.45062
North America	2012	0.02486	0.12014
Australia	2012	0.00363	0.04636
Southeast Asia	2012	0.0157	0.08728
South America	2012	0.00192	0.02758
South Asia	2012	0.43899	0.45469
Middle East	2012	0.00399	0.04044
Africa	2012.25	0	0
East Asia	2012.25	0.00454	0.05502
Europe	2012.25	0.07815	0.24545
North America	2012.25	0.01437	0.10595
Australia	2012.25	0.00182	0.04079
Southeast Asia	2012.25	0.10495	0.26895
South America	2012.25	0.002	0.04468
South Asia	2012.25	0.79038	0.36907
Middle East	2012.25	0	0
Africa	2012.5	0.00037	0.00834
East Asia	2012.5	0.00138	0.02649
Europe	2012.5	0.03957	0.16261
North America	2012.5	0.01758	0.11425
Australia	2012.5	0.0022	0.03531
Southeast Asia	2012.5	0.01042	0.09102

South America	2012.5	0.00155	0.03464
South Asia	2012.5	0.91502	0.24695
Middle East	2012.5	0.0016	0.02401
Africa	2012.75	0	0
East Asia	2012.75	0.00181	0.03602
Europe	2012.75	0.02206	0.12467
North America	2012.75	0.01327	0.09556
Australia	2012.75	0	0
Southeast Asia	2012.75	0.00078	0.01128
South America	2012.75	0	0
South Asia	2012.75	0.9655	0.15024
Middle East	2012.75	0.00122	0.02173
Africa	2013	0	0
East Asia	2013	0	0
Europe	2013	0.02264	0.12874
North America	2013	0.05751	0.20791
Australia	2013	0.00109	0.02448
Southeast Asia	2013	0.00011	0.00247
South America	2013	0.00866	0.08921
South Asia	2013	0.90851	0.265
Middle East	2013	0.00316	0.04961
Africa	2013.25	0.00012	0.0026
East Asia	2013.25	0.01297	0.08895
Europe	2013.25	0.09115	0.22745
North America	2013.25	0.01885	0.10945
Australia	2013.25	0.02837	0.1325
Southeast Asia	2013.25	0.00762	0.05233
South America	2013.25	0.0007	0.01556
South Asia	2013.25	0.84943	0.29163
Middle East	2013.25	0.00215	0.02695

Africa	2013.5	0.00058	0.013
East Asia	2013.5	0.04353	0.17253
Europe	2013.5	0.11351	0.2628
North America	2013.5	0.0241	0.11818
Australia	2013.5	0.32924	0.37021
Southeast Asia	2013.5	0.07475	0.22367
South America	2013.5	0	0
South Asia	2013.5	0.39486	0.38613
Middle East	2013.5	0.00569	0.05606
Africa	2013.75	0	0
East Asia	2013.75	0.11457	0.28498
Europe	2013.75	0.00239	0.03574
North America	2013.75	0.26325	0.36182
Australia	2013.75	0.59065	0.42814
Southeast Asia	2013.75	0.01859	0.10515
South America	2013.75	0	0
South Asia	2013.75	0.00151	0.02763
Middle East	2013.75	0.00252	0.03986
Africa	2014	0	0
East Asia	2014	0.6116	0.47659
Europe	2014	0.02579	0.12507
North America	2014	0.20122	0.36126
Australia	2014	0.03989	0.17286
Southeast Asia	2014	0.00747	0.06817
South America	2014	0.00135	0.02789
South Asia	2014	0.00089	0.02054
Middle East	2014	0.1171	0.27199
Africa	2014.25	0.00072	0.02021
East Asia	2014.25	0.06659	0.21016
Europe	2014.25	0.00559	0.05712

North America	2014.25	0.02768	0.13454
Australia	2014.25	0.3479	0.45986
Southeast Asia	2014.25	0.02141	0.11597
South America	2014.25	0.00392	0.05567
South Asia	2014.25	0.00182	0.03328
Middle East	2014.25	0.52553	0.49365
Africa	2014.5	0	0
East Asia	2014.5	0.00423	0.05107
Europe	2014.5	0.00385	0.04167
North America	2014.5	0.02812	0.14754
Australia	2014.5	0.62567	0.47801
Southeast Asia	2014.5	0.00234	0.0404
South America	2014.5	0.03648	0.15294
South Asia	2014.5	0.00109	0.02771
Middle East	2014.5	0.29092	0.43938

Supplementary Table 7. Statistical supported diffusion rates of global epidemiology links between worldwide localities during each epidemic season. Geographical locations are coded as follows: Af=Africa; AUS=Australia; EA=East Asia; EU=Europe; NAm=North America; SEA=Southeast Asia; SAm=South America; and SA=South Asia.

Migration from	Mean actual rate	Mean indicator	Bayes Factor
**2009–2010**			
SEA to NAm	2.244	1	>1000
SEA to AUS	1.839	1	>1000
NAm to EU	1.628	1	>1000
SEA to ME	1.240	1	>1000
SEA to EA	2.062	1	>1000
EU to AF	0.882	0.978	100–1000
EU to SEA	0.790	0.920	3–100
NAm to SAm	0.844	0.899	3–100
SEA to EU	1.497	0.871	3–100
N to AF	0.715	0.848	3–100
EA to N	0.943	0.805	3–100
AUS to NAm	0.73	0.696	3–100
EA to SEA	0.689	0.655	3–100
SAm to AUS	0.750	0.600	3–100
SEA to SAm	0.652	0.586	3–100
**2010–2011**			
EU to NAm	3.094	1	>1000
EU to SAm	1.731	1	>1000
EU to ME	0.591	1	>1000
SEA to EA	2.523	1	>1000
SEA to EU	2.934	1	>1000

EU to AF	1.168	0.998	>1000
SEA to NAm	1.183	0.993	>1000
EU to AUS	0.91	0.988	100–1000
SAm to SEA	0.568	0.967	100–1000
EU to SA	0.875	0.961	100–1000
EU to EA	0.849	0.948	100–1000
NAm to EA	0.551	0.93	3–100
EA to AF	0.837	0.925	3–100
EA to NAm	0.542	0.879	3–100
EA to SEA	0.740	0.646	3–100
SEA to AUS	0.760	0.595	3–100
EA to AUS	0.705	0.590	3–100
**2011–2012**			
SEA to EA	1.432	0.999	>1000
NAm to SAm	1.504	0.999	>1000
EU to ME	0.857	0.998	>1000
SA to AF	1.309	0.996	>1000
NAm to AUS	0.992	0.994	>1000
SAm to NAm	0.790	0.990	100–1000
NAm to EU	1.639	0.988	100–1000
SA to NAm	2.290	0.968	100–1000
SA to EU	1.535	0.964	100–1000
SA to SEA	1.417	0.944	100–1000
SAm to EU	0.584	0.836	3–100
SEA to AUS	0.784	0.715	3–100
SA to AUS	0.673	0.592	3–100
**2012–2013**			
EU to NAm	2.771	1	>1000
EU to SA	1.314	1	>1000
EU to AF	1.518	1	>1000

NAm to AUS	1.614	1	>1000
NAm to EA	1.945	0.999	>1000
EU to ME	0.609	0.998	>1000
SAm to NAm	0.878	0.98	100–1000
EU to SAm	1.234	0.974	100–1000
NAm to SAm	1.007	0.967	100–1000
EU to AUS	0.857	0.957	100–1000
NAm to SEA	1.065	0.956	100–1000
SEA to EU	1.570	0.897	3–100
EA to EU	0.639	0.779	3–100
EU to EA	0.761	0.739	3–100
**2013–2014**			
AUS to NAm	0.980	0.988	100–1000
EA to SEA	0.950	0.941	3–100
EU to SA	1.021	0.792	3–100
ME to SEA	0.931	0.866	3–100
NAm to ME	0.844	0.807	3–100
ME to NAm	1.080	0.577	3–100
ME to EA	0.980	0.600	3–100
EA to AF	0.945	0.670	3–100
ME to AUS	0.801	0.588	3–100
EU to SEA	0.790	0.562	3–100
EU to NAm	0.800	0.594	3–100
EA to ME	0.649	0.676	3–100
SA to NAm	0.527	0.512	3–100

Supplementary Table 8. Comparison of transition state counts of H1N1/2009 virus migration for nine individual geographical regions in each epidemic season.

Location	Migration	2009–2010	2010–2011	2011–2012	2012–2013	2013–2014
**Africa**	Out	0.000	0.770	0.000	1.887	2.884
	In	7.054	14.517	9.634	17.175	9.353
**Australia**	Out	2.016	0.627	0.263	0.508	6.577
	In	15.940	13.480	9.455	22.297	30.852
**East Asia**	Out	8.710	13.931	0.501	3.082	43.580
	In	16.853	33.017	5.301	20.278	16.270
**Europe**	Out	9.180	71.240	4.665	98.988	30.530
	In	20.284	29.218	21.068	11.107	32.670
**Middle East**	Out	-	0.550	0.000	0.192	15.643
	In	-	4.181	3.058	6.183	19.350
**North America**	Out	19.006	3.815	24.120	41.456	76.480
	In	22.794	38.818	21.170	38.153	13.490
**South America**	Out	1.712	2.619	5.621	5.345	0.726
	In	7.909	13.951	8.084	20.309	22.527
**South Asia**	Out	0.376	0.260	47.262	2.079	1.387
	In	9.912	6.920	1.132	15.295	10.240
**Southeast Asia**	Out	65.530	67.847	7.995	8.489	1.995
	In	5.869	5.184	11.649	10.828	25.020

Supplementary Table 9. The estimated TMRCAs of each gene segment in different years, 2009–2014. The temporal analyses were performed in BEAST using a global dataset of 485 H1N1/2009 sequences.

Gene	Mean TMRCA	Upper 95% HPD	Lower 95% HPD
**2009**			
PB2	2008.918	2008.971	2009.151
PB1	2008.977	2008.881	2009.739
PA	2008.855	2008.871	2009.091
HA	2009.032	2008.921	2009.130
NP	2009.039	2008.888	2009.157
NA	2009.080	2008.981	2009.181
MP	2008.848	2008.951	2009.191
NS	2008.900	2007.931	2009.171
**2010**			
PB2	2008.918	2008.971	2009.151
PB1	2008.977	2008.881	2009.739
PA	2008.855	2008.871	2009.091
HA	2009.318	2009.271	2009.328
NP	2009.322	2009.271	2009.361
NA	2009.296	2009.221	2009.361
MP	2009.095	2009.231	2009.371
NS	2009.160	2009.161	2009.361
**2011**			
PB2	2009.831	2009.578	2010.079
PB1	2009.014	2009.025	2009.049
PA	2009.059	2009.179	2009.202
HA	2009.608	-	2014.921
NP	2009.355	-	2014.921
NA	2009.332	-	2014.921
MP	2009.095	2009.231	2009.371

NS	2009.160	-	2014.921
**2012**			
PB2	2010.281	2010.158	2010.439
PB1	2010.127	2009.936	2010.323
PA	2010.105	2010.012	2010.306
HA	2010.220	2010.037	2010.382
NP	2010.169	2009.931	2010.351
NA	2010.457	2010.401	2010.091
MP	2010.402	2010.551	2010.631
NS	2010.132	2010.101	2010.471
**2013**			
PB2	2010.281	2010.158	2010.439
PB1	2010.127	2009.936	2010.323
PA	2010.105	2010.012	2010.306
HA	2010.220	2010.037	2010.382
NP	2010.169	2009.931	2010.351
NA	2010.457	2010.401	2010.091
MP	2010.402	2010.551	2010.631
NS	2010.132	2010.101	2010.471
**2014**			
PB2	2013.169	2013.041	2013.255
PB1	2013.085	2013.011	2013.246
PA	2013.105	2012.931	2013.203
HA	2013.103	2012.897	2013.215
NP	2013.177	2013.114	2013.255
NA	2013.214	2013.086	2013.351
MP	2013.287	2013.291	2013.341
NS	2012.062	2011.941	2012.441
